# Hollow organic capsules assemble into cellular semiconductors

**DOI:** 10.1038/s41467-018-04246-0

**Published:** 2018-05-16

**Authors:** Boyuan Zhang, Raúl Hernández Sánchez, Yu Zhong, Melissa Ball, Maxwell W. Terban, Daniel Paley, Simon J. L. Billinge, Fay Ng, Michael L. Steigerwald, Colin Nuckolls

**Affiliations:** 1 Department of Chemistry, Columbia University, New York, NY 10027 USA; 2 Columbia Nano Initiative, Columbia University, New York, NY 10027 USA; 3 Department of Applied Physics and Applied Mathematics, Columbia University, New York, NY 10027 USA; 4 Condensed Matter Physics and Materials Science Department, Brookhaven National Laboratory, Upton, NY 11973 USA; 5 The State Key Laboratory of Refractories and Metallurgy, Institute of Advanced Materials and Nanotechnology, School of Chemistry and Chemical Engineering, Wuhan University of Science and Technology, Wuhan, 430081 China

## Abstract

Self-assembly of electroactive molecules is a promising route to new types of functional semiconductors. Here we report a capsule-shaped molecule that assembles itself into a cellular semiconducting material. The interior space of the capsule with a volume of ~415 Å^3^ is a nanoenvironment that can accommodate a guest. To self-assemble these capsules into electronic materials, we functionalize the thiophene rings with bromines, which encode self-assembly into two-dimensional layers held together through halogen bonding interactions. In the solid state and in films, these two-dimensional layers assemble into the three-dimensional crystalline structure. This hollow material is able to form the active layer in field effect transistor devices. We find that the current of these devices has strong response to the guest’s interaction within the hollow spaces in the film. These devices are remarkable in their ability to distinguish, through their electrical response, between small differences in the guest.

## Abstract (web summary)

Perylene diimide-bithiophene macrocycles are electroactive and shape-persistent hosts. Here, the authors describe their self-assembly into a cellular organic semiconducting film whose voids are electrically sensitive to different guests, and which can function as the active layer in a field-effect transistor device.

## Introduction

There is a growing class of electroactive, conjugated cyclic molecules that are being applied in several areas of materials science^1–27^. These cyclic, conjugated organic semiconductors have interior spaces that should be useful as a locus for guest inclusion to tune the electronic and optoelectronic properties^6,22,28–30^. Conjugated, cyclic semiconductors that incorporate diphenyl perylene diimides (PDIs) have many benefits as the active elements in organic field-effect transistors (OFETs), organic photovoltaics^31^, and organic photodetectors^32^. In the cases we have studied until now, the diphenyl–PDI subunits provide open macrocycles with cavity diameters so large that they exhibit dynamic stereochemistry as the PDI subunits readily rotate through the interior of the ring^20^.

**Fig. 1:**
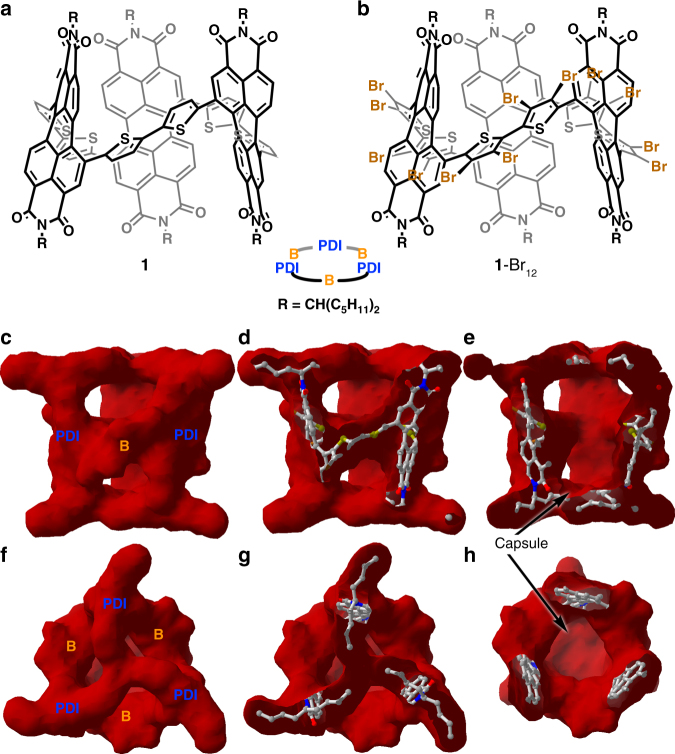
Structures of cellular semiconductors. **a** Trimer **1**; and **b** **1**-Br_12_. Van der Waals Surface of **1**-Br_12_ seen from the side (**c**), and top (**f**). In the sequence **c-e** and **f**–**h** the molecule is trimmed down to expose its cavity or capsule

We describe here a new electronic material whose molecular components are shape persistent and can be functionalized so they self-assemble into semiconducting films. These films feature regular open spaces, cells, in them. We call this new material a cellular organic semiconductor. The molecular substructure results from the union of bithiophenes (B) and PDI into a macrocycle. We fully utilize the interior of the macrocycle in devices, and endow the materials with permanent, open voids, by rigidifying the macrocycle so they cannot collapse. We describe the syntheses and structures of the organic semiconductors shown in Fig. 1a, b: the trimeric macrocycle **1**, (-PDI-B-)_3_ and its brominated version **1**-Br_12_. These macrocycles exist as a single pair of enantiomers and are shape persistent to temperatures above 160 °C. Their capsular structure is capped on the ends by the alkyl sidechains and on the equator by the electronic components (the PDI and B subunits). This is shown in Fig. 1c–h. While **1** and **1**-Br_12_ share the same overall shape and molecular structure, compound **1** does not organize well in films or in the solid state. However, the twelve bromines of **1**-Br_12_ participate in halogen bonding interactions, facilitating self-assembly in films and crystals. **1**-Br_12_’s self-organization creates films that have regular voids in them, forming the hollow organic semiconducting phase. The remarkable finding is that **1-**Br_12_’s cellular films act as the active layer in OFETs, and the electrical response depends on the guest that occupies the interior space.

## Results

### Capsule construction

We developed a synthesis of **1** based on our own previous studies to make the diphenyl-PDI macrocycles^20^ that builds from the methodology originally pioneered for cyclothiophenes and later for cycloparaphenylenes^20,31^. The Supporting Information contains the details of the syntheses and characterization (Supplementary Methods and Supplementary Fig. 1–3) of **1** and **1**-Br_12_. For each of the macrocycles, the 1,7-dithienyl-PDI subunit introduces an element of chirality because it can exist in either a *R*- or *S*-helical conformation. This allows for the possibility of two pairs of enantiomers (RRR/SSS and RRS/SSR)^20^. However, in the reaction to form **1**, we only observe the *RRR*/*SSS* pair. We introduce bromine atoms in the thiophene rings of **1** to encourage self-assembly through halogen bonding interactions^33^.

To test the shape persistence of these macrocycles, we separate the two enantiomers of **1**, using a chiral stationary phase for HPLC, and monitor their interconversion as we heat the samples. The two enantiomers of **1** exhibit an intense (and opposite) chiroptic response in their circular dichroism spectra (Supplementary Fig. 2). Remarkably, the enantiomers of **1** do not interconvert, even when heated up to 160 °C. Macrocycle **1** is the first PDI-based cyclic semiconductor that can be isolated in its optically active form and is shape-persistent.

### Capsule structure

**Fig. 2:**
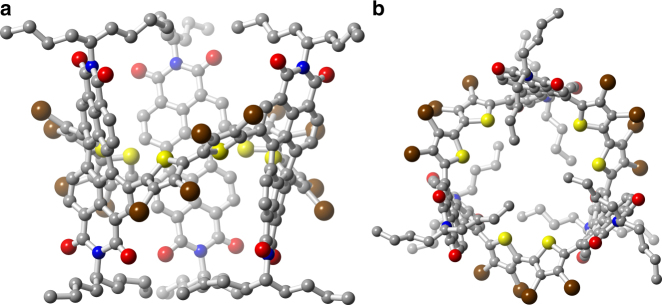
Molecular structure from SCXRD of **1**-Br_12_. **a** Side and **b** top view of (*SSS*)-**1**-Br_12_. C, N, O, S, and Br atoms are colored in gray, blue, red, yellow, and brown, respectively. Hydrogen atoms have been removed to clarify the view. The alkyl chains on the imide are refined to only nine of the eleven carbon atoms due to disorder (see Supplementary Methods)

**Fig. 3:**
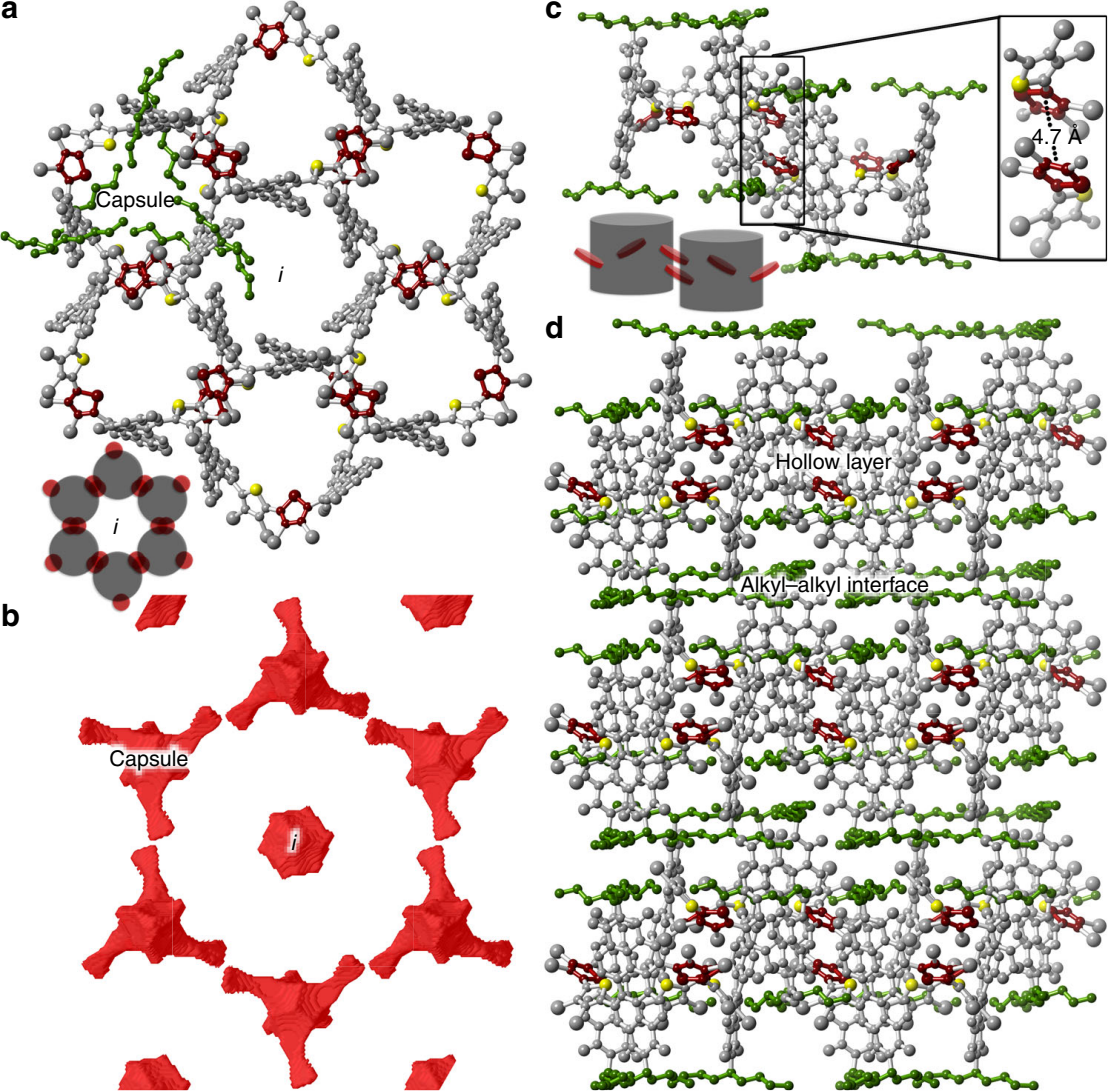
Structural packing of **1**-Br_12_. **a** View of the honeycomb structure in the ab plane for **1**-Br_12_. The capsule and *i* corresponds to the internal cavity of **1**-Br_12_ and the cavity formed by the packing of **1**-Br_12_, respectively. The remaining sulfur atoms are colored in yellow to provide a marker to identify the macrocycle cavities. See bottom left cartoon. Highlighted in green are the imide side chains (some of the sidechains have been removed to clarify the view of the cavity). In red are the thiophene rings likely involved in holding the macrocycles together. **b** Surface map of the void space in the ab plane of **1**-Br_12_. **c** Two molecules of **1**-Br_12_ where the thiophene-to-thiophene interaction is highlighted as an inset. Bottom left cartoon represents this interaction. **d** View of the packing of **1**-Br_12_. As shown, the vertical stacking follows the *c* axis. The alkyl sidechains of the imide are shown in green. Hydrogen atoms have been removed from all structures to clarify the view

The crystals of **1**, while sizable and faceted, do not diffract well enough to yield a structure from single-crystal X-ray diffraction (SCXRD), but we were able to grow single crystals of sufficient quality to yield the structures of **1**-Br_12_. Figure 2a, b displays the structure of one of the two enantiomers of **1**-Br_12_ [(*SSS*)-**1**-Br_12_]. Both enantiomers of **1**-Br_12_ are present in the crystalline state. The structure of **1-**Br_12_ is cylindrical with the three sets of bithiophenes and three PDIs forming the walls at the equator. The ends of the cylinder are capped with branched alkyl chains (Fig. 2 and highlighted in green in Fig. 3a). This creates windows on the side of the structure, displayed in Figs. 1c, f and 2a. We estimate the interior volume of the capsule in **1-**Br_12_ (shown in Fig. 1e, h) to be approximately 415 Å^3^^34^.

### Cellular solids from capsular nanostructures

The packing structure in the solid state of **1**-Br_12_ (Fig. 3) reveals why the bromines were necessary for long range crystallinity. The structure is composed of sheets of a honeycomb-like arrangement in the ab plane (Fig. 3a). The interactions that bind the cylinders within the ab plane are from neighboring brominated thiophene rings (4.7 Å apart, marked in red in Fig. 3a, c–d) and some secondary *π*–*π* contacts between those same rings (marked in blue in Supplementary Fig. 4). The PDIs do not *π*-stack with each other. Instead halogen bonding from the functionalized thiophenes drives the self-assembly process. The cavity of **1**-Br_12_ (labeled “Capsule” in Fig. 3a, b) is ~11.4 Å in diameter and is a three-fold symmetric chiral nanoenvironment for guest incorporation within the two-dimensional layer. Due to the packing of the subunits of **1**-Br_12_ into a hexameric cyclic structure, a second cavity forms at the center of each hexagon (labeled *i* in Fig. 3a, b). This cavity is not elongated compared to the capsule’s cavity, and each one accounts for ~110 Å^3^ (Supplementary Fig. 5). These honeycomb two-dimensional, cellular sheets then stack through the packing of the alkyl side chains of the imides, (shown in green in Fig. 3). This packing arrangement propagates along the *c* axis, hinting that these materials could likely be exfoliated to yield molecularly thin sheets of **1**-Br_12_.

We find that **1-**Br_12_ self-assembly in cast thin films and powder samples is analogous to what we described above for the single crystal. Supplementary Figs 6, 7 compare the thin film and powder diffraction data for **1-**Br_12_ with the simulated pattern calculated from the SCXRD data. No other reflections are present in the films or powders indicating that the self-assembly motif using the halogen bonding is robust. In order to extract quantitative information about the long-range crystallinity of these new hollow semiconductors we performed pair distribution function (PDF) analyses on powders of **1** and **1**-Br_12_ (Supplementary Figs. 8–12). Supplementary Fig. 12 displays the PDF of **1**-Br_12_ at low (1.81 Å^−1^) and high (6.33 Å^−1^) *Q*_max_ to give a visualization of the coherence length. The lower and upper bounds of the coherence length of **1**-Br_12_ are 335 and 385 Å, respectively (Supplementary Fig. 12). To put these values in perspective, this indicates that crystalline domains within **1**-Br_12_ contain on average up to 23 to 27 capsules arranged in one direction. In contrast, **1**, with poor crystallinity and an inability to self-organize (Supplementary Fig. 6), is essentially amorphous. The analysis shows coherence lengths ranging from 65 to 120 Å (Supplementary Fig. 11), which is only a few capsules in length.

### Electron transport through cellular films

**Fig. 4:**
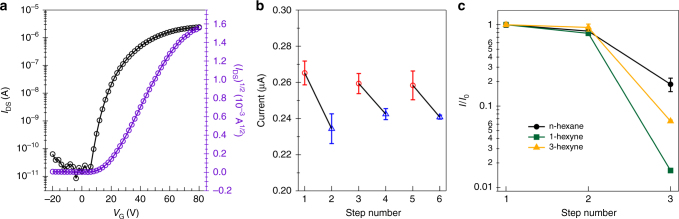
Electron transport for cellular films. **a** Transfer characteristics of OFET device for **1**-Br_12_. **b** Device cycling response under vacuum (red circles) and N_2_ atmosphere (blue triangles). **c** Normalized behavior of the device response under vacuum (step 1), N_2_ (step 2), and different analytes atmosphere (step 3: n-hexane, 3-hexyne and 1-hexyne). Error bars represent the standard error obtained in three measurements

Figure 4a displays transfer curves from an OFET constructed using a self-assembled thin film of **1**-Br_12_. Details for the device dimensions and its properties can be found in the Supplementary Methods. The device exhibits electron transporting character and has a mobility of ~1.5 × 10^−2^ cm^2^ V^−1^ s^−1^. The mobility of **1**-Br_12_ is more than 20 times greater than that of **1** (~6.8 × 10^−4^ cm^2^ V^−1^ s^−1^) (Supplementary Fig. 13). We attribute this to the robust self-assembly process for **1**-Br_12._ From atomic force microscope height images, films of **1** display a smooth surface with root mean square (RMS) roughness of 0.347 nm; in contrast **1**-Br_12_ displays a larger RMS roughness of 3.2 nm, presumably due to its more crystalline nature and better self-assembly properties (Supplementary Fig. 14).

The exciting finding is that the devices show modulation in the drain current depending on what, if anything, is within the voids in the cellular film. Details of the experimental setup to measure the exposure to gases are given in the Supplementary Methods. Every potential guest tested had a measurable effect on the drain current in the device, but the absolute levels of the drain current varied depending on the guest. Figure 4b is a representative data set for a device measured sequentially in steps as the atmosphere is changed between vacuum and N_2_. In certain cases, traditional OFETs show a differential response to nitrogen and vacuum that arises from extrinsic effects (moisture, oxygen, or dielectric effects)^35,36^. We can test the importance of the cellular semiconductor’s response by making a control FET device from **1**, which does not self-organize into cellular semiconducting films or crystals. We observe no response when comparing its response to nitrogen and vacuum (Supplementary Fig. 15a).

Incorporating more polarizable and functional guests causes more pronounced changes in the drain current. Supplementary Fig. 15b compares the effects of several different guests with a variety of functional groups such as ketones, alcohols, nitriles, alkynes, and alkanes. In each case we are able to differentiate the guest by the current in the device. We highlight here one striking example of how this material responds to a series of closely related hydrocarbons. These hydrocarbons were chosen so that their length, size, and polarity were roughly similar to that of n-hexane, while having an additional functional group in them. Fig. 4c compares the transistor output for three devices exposed to n-hexane, 3-hexyne, or 1-hexyne, carried by nitrogen under their saturated vapor pressure (148, 99, and 138 mmHg at 25 °C, respectively)^37^. Remarkably, the devices can easily detect these three materials and distinguish them from one another. There is also specificity towards particular analytes; the trend in the device responses does not simply follow the vapor pressure. For n-hexane and 3-hexyne the data was collected after the OFET was in contact with the vapor for ~70 min (Supplementary Fig. 16a). After this time, the drain current no longer decreased. The original current levels for n-hexane and 3-hexyne could be restored by placing the devices in vacuum (Supplementary Fig. 16b). During these long exposures, we speculate that the guests infiltrate the films to reside in the active part of the films at the gate dielectric interface^38,39^.

We tested the sensitivity of these hollow films and find that there is a linear response between vacuum and 20 part per thousand of the analyte in the atmosphere, after we observed a plateau region as the concentration reached saturation (Supplementary Fig. 17). Access to the porous network formed by the capsules interior (~415 Å^3^) and the *i*-sites (~110 Å^3^) is granted by the windows in the cellular structure as shown in Fig. 1c–h. Using BET, we find that powders of **1**-Br_12_ have a surface area of 20 m^2^ g^−1^ (versus 1.2 m^2^ g^−1^ for **1**). As n-hexane has a van der Waals molecular volume of ~113 Å^3^^40^, the implication is that n-hexane would not fit into the *i*-site. In addition, most of the cellular nature of self-assembled **1**-Br_12_ comes from the capsule interior which is present in a 2:1 numerical ratio relative to the *i*-site. Given the 8-fold difference in volume between these two cavities, we speculate that guests can be accommodated most feasibly at the capsules’ interior.

1-hexyne behaves differently than each of the other guests tested. For the devices in an atmosphere of 1-hexyne, the current continues to drop and does not reach a plateau even at times that exceed 2 h of exposure. In addition, the 1-hexyne devices do not recover to their original levels when placed in vacuum (Supplementary Fig. 18). We speculate that the terminal alkyne is undergoing a reaction under the device conditions that is not possible with the internal alkyne or the alkane. This offers the intriguing possibility that in addition to sensing, the nanoenvironments in these hollow semiconductors can be used as nanoreactors.

## Discussion

We have described here a shape persistent, hollow macrocycle that self-assembles both in the solid state and thin films to form cellular organic semiconductors. The macrocycle is chiral and conformationally locked into a capsular structure, with ~415 Å^3^ volume within its interior. The self-assembly of a brominated derivative of the trimer into cellular films forms the active layer in an organic field-effect transistor device. Once assembled, these films have periodic, nanoscopic, cellular voids. Because the macrocyclic component in the film is conformationally locked, the self-assembled films maintain their interior, open spaces. The hollow films of **1**-Br_12_ are responsive to the atmosphere in which the OFETs are measured. These studies chart a clear path to using the interior of the cellular organic semiconductors as gas sensors^41,42^ and nanoreactors.

### Data availability

All data are available from the authors upon reasonable request. The X-ray crystallographic coordinates for structures reported in this study have been deposited at the Cambridge Crystallographic Data Centre (CCDC), under deposition number 1834626. These data can be obtained free of charge from The Cambridge Crystallographic Data Centre via www.ccdc.cam.ac.uk/data_request/cif.

## Electronic supplementary material

Supplementary Information
