# Supplementary material for: Hollow organic capsules assemble into cellular semiconductors
Source: Nat Commun. 2018 May 16;9:1957. doi: 10.1038/s41467-018-04246-0 (PMC5956104; doi:10.1038/s41467-018-04246-0)
Supplement: Supplementary file 1 — Supplementary Information [file 41467_2018_4246_MOESM1_ESM.pdf]

**Supplementary Materials For:**

# Hollow Organic Capsules Assemble into Cellular Semiconductors

Zhang et al.

## 1. UV-Vis, CD, and cyclic voltammetry

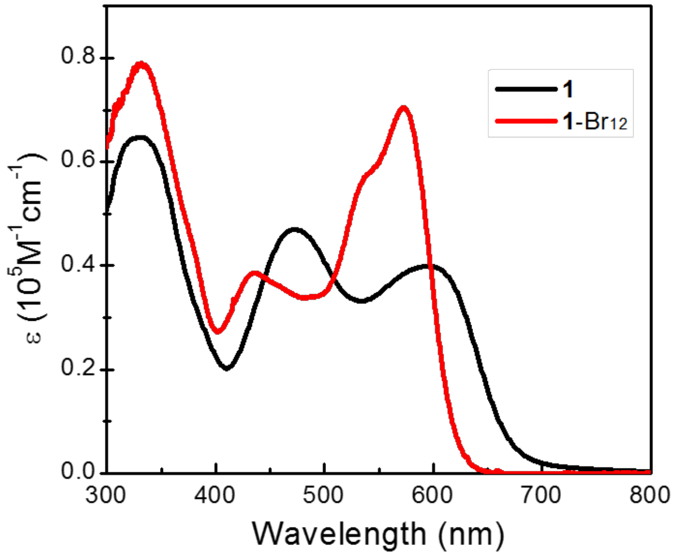


**Supplementary Figure 1.** UV-vis absorption spectra of **1** and **1**-$Br_{12}$. (1 x $10^{-5}$ M concentration in dichloromethane with a path length l = 1cm).

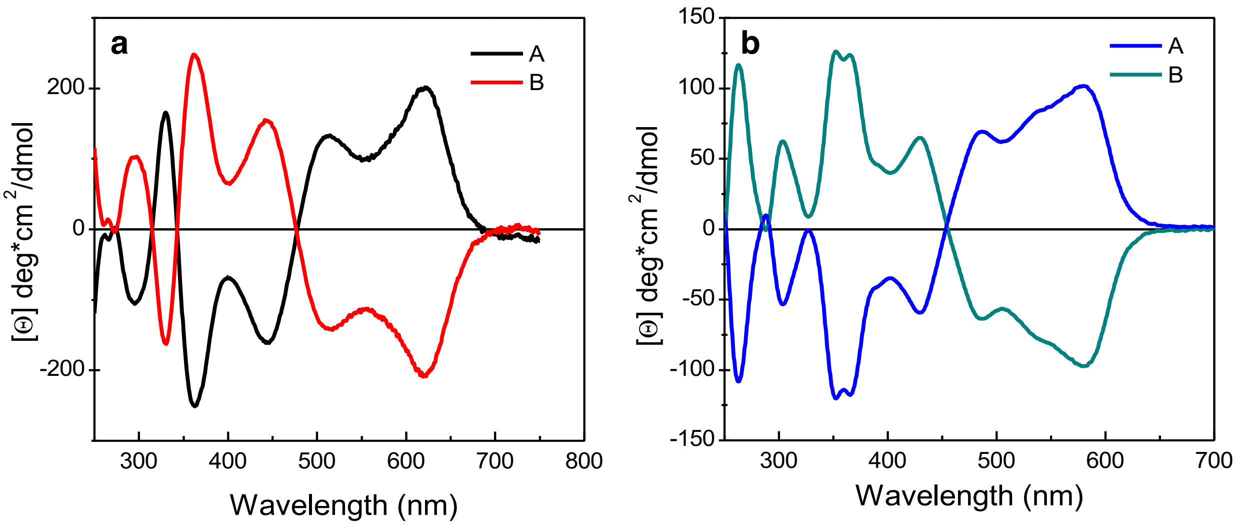


**Supplementary Figure 2.** CD of (a) (+)-**1** (black trace) and (–)-**1** (red trace) and (b) (+)-**1**-$Br_{12}$ (teal trace) and (–)-**1**-$Br_{12}$ (blue trace).

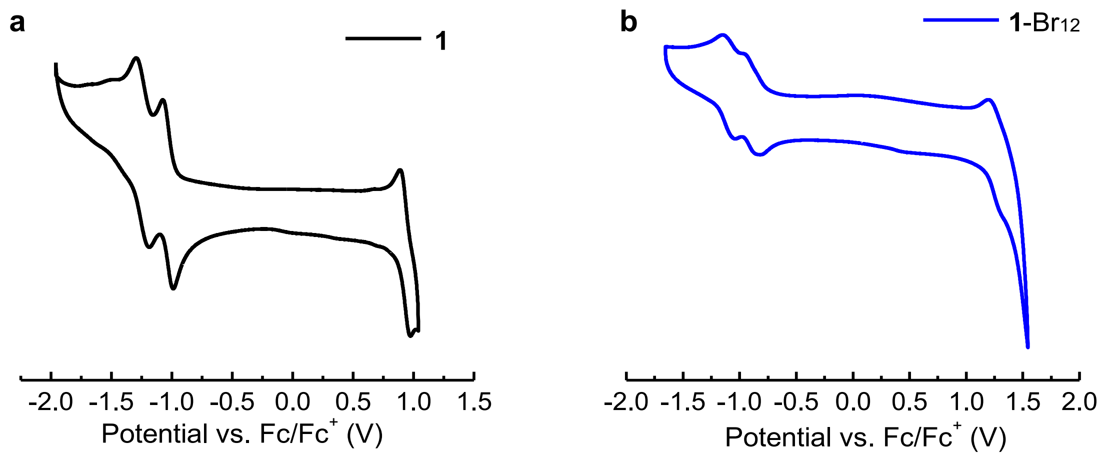


**Supplementary Figure 3.** Cyclic voltammograms for **a, 1**(black) and **b, 1**-$Br_{12}$ (blue).

**Supplementary Table 1.** Reversible reduction potentials of **1** and **1**-$Br_{12}$ with reference to $Fc/Fc^+$ and their optical bangaps.

| **Compound** | $\mathbf{E}_{\mathbf{red}}{}^{a}$ (eV) | $\mathbf{E}_{oxi}{}^{a}$ (eV) | Optical $\mathbf{E}_{gap}$ (eV) |
|---|---|---|---|
| **1** | −1.03 | 0.93 | 1.84 |
| **1**-$Br_{12}$ | −0.90 | 1.26 | 2.00 |

[a]Reduction and oxidation potentials were estimated from half-wave potential ($E_{1/2}$).

## 2. PXRD and SCXRD

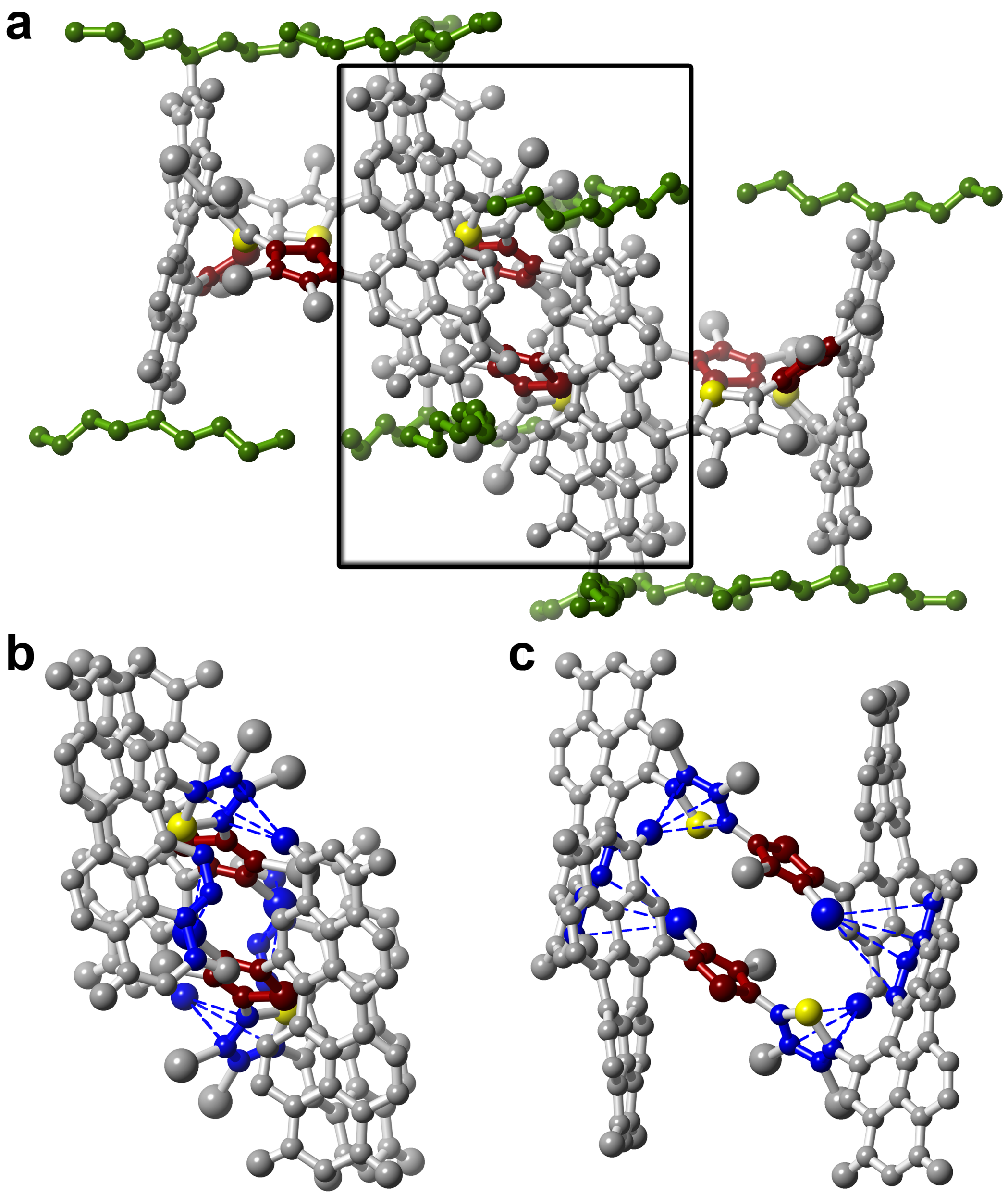


**Supplementary Figure 4. a**, Image displaying two molecules of **1**-$Br_{12}$ with the thiophene-to-thiophene interaction enclosed within a black rectangle. **b**, Picture displaying the additional interactions in blue: 1) O-thiophene, d = 3.13 to 3.63 Å; and 2) Br-PDI, d = 3.49 to 3.98 Å. View corresponds to the black rectangle in **a** after removing all atoms exept the brominated bithiophenes involved in the interaction and the neighboring PDIs. **c**, Image in **b** rotated by 90 degrees to better appreciate the additional interactions highlighted in blue.

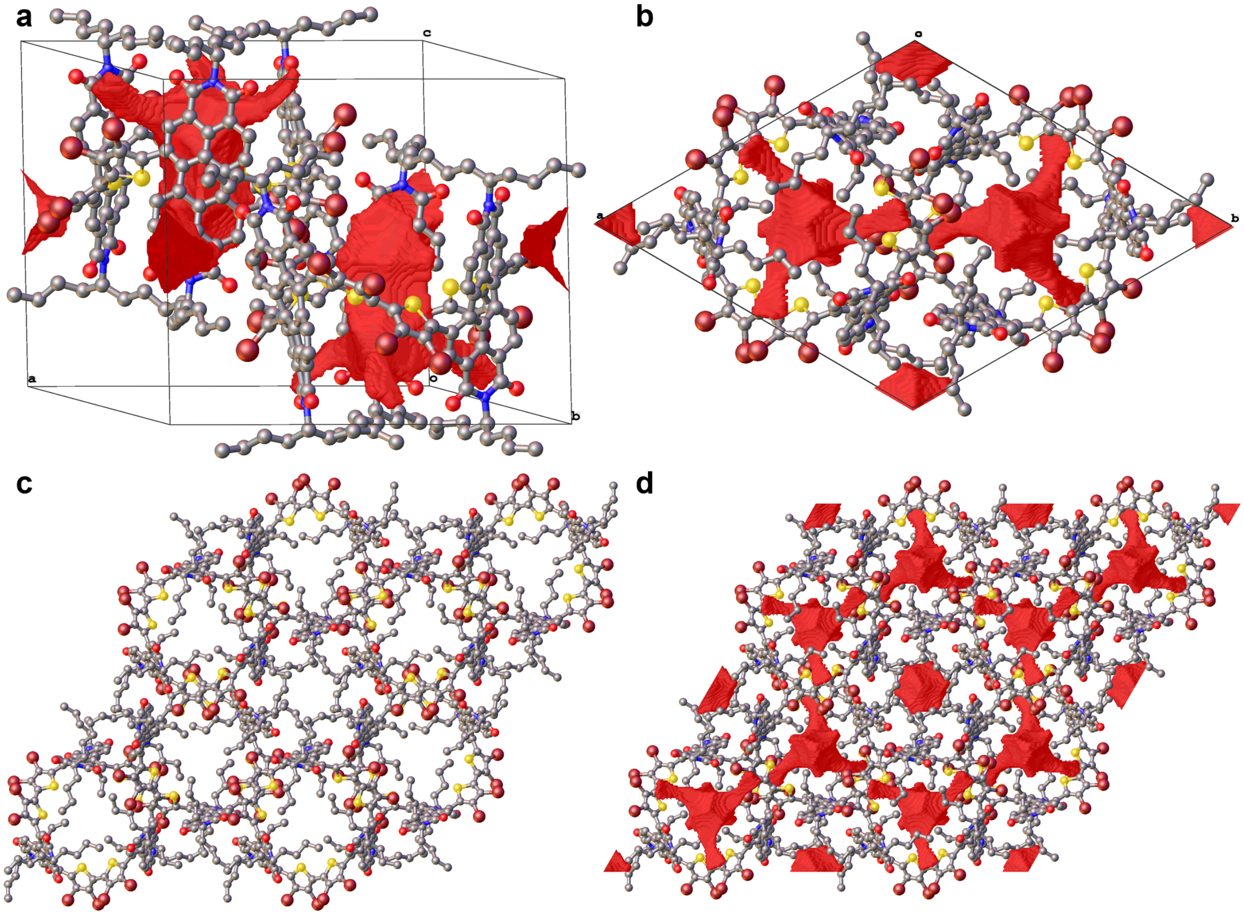


**Supplementary Figure 5.** Single crystal structure of **1**-$Br_{12}$. **a**, Side and **b**, top view of the unit cell of **1**-$Br_{12}$. **c**, The structural packing of **1**-$Br_{12}$ is shown viewing down the c-axis. **d**, The same view as in **c** with addition of the surface map (shown in red) of the void space in **1**-$Br_{12}$.

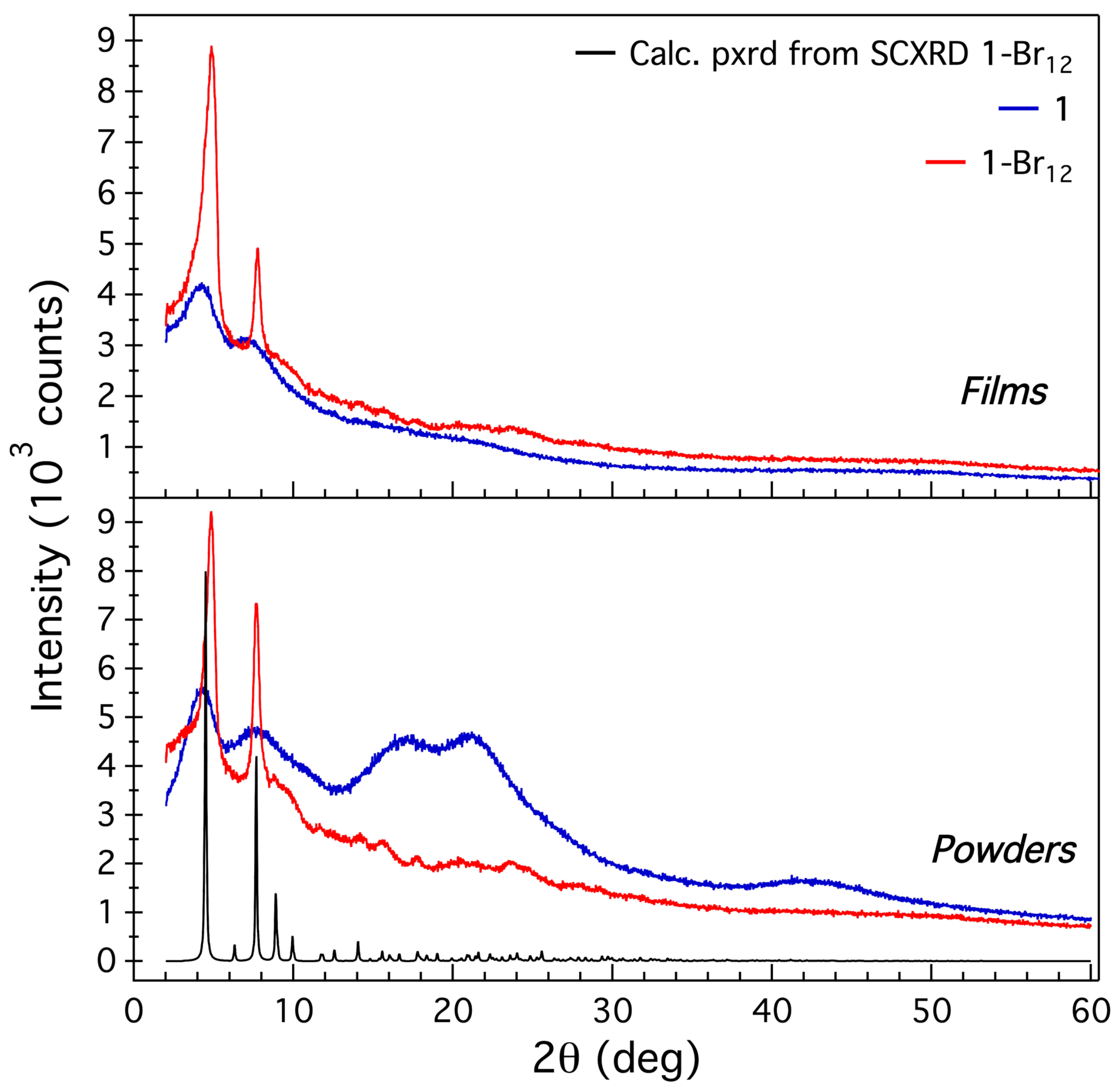


**Supplementary Figure 6.** Powder X-ray diffraction (PXRD) of **1** and **1**-$Br_{12}$. The top panel corresponds to films drop-cast from a chloroform solution, while the bottom panel is data obtained from powder samples. As a reference, the black line represents the predicted PXRD pattern obtained from the single crystal data of **1**-$Br_{12}$.

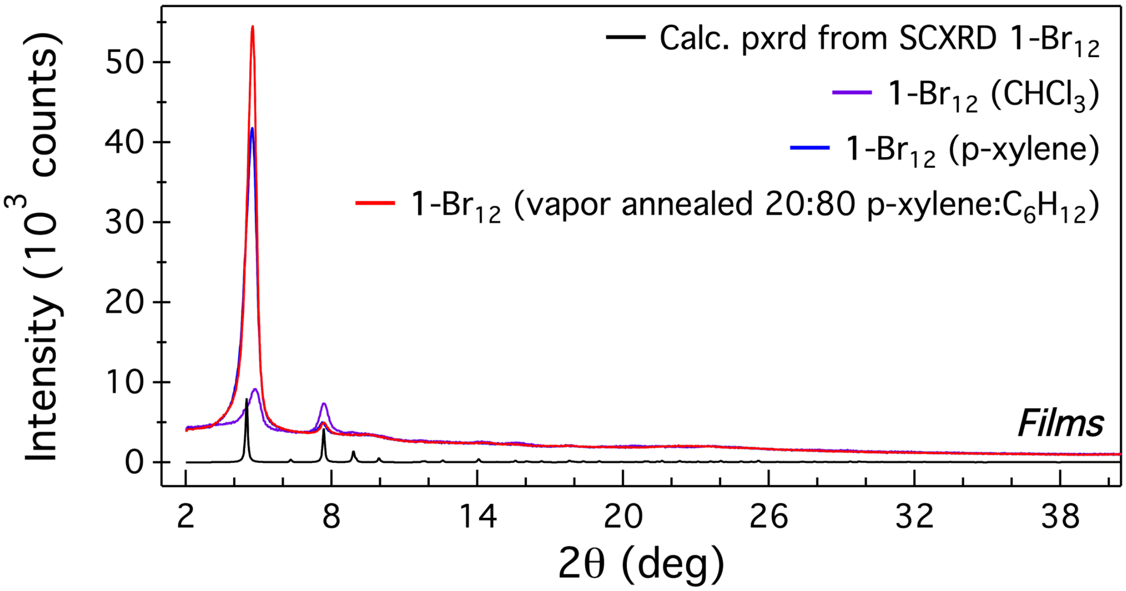


**Supplementary Figure 7.** Powder X-ray diffraction (PXRD) of drop-cast films of **1**-$Br_{12}$. The patterns of the as-deposited film obtained for **1**-$Br_{12}$ when dissolved in chloroform or p-xylene are shown by the purple and blue traces, respectively. Furthermore, an increase in polycrystallinity is observed for the film obtained from p-xylene when this is vapor annealed at 70 °C in 20:80 p-xylene:cyclohexane (red trace). As a reference, the black line represents the predicted PXRD pattern obtained from the single crystal data of **1**-$Br_{12}$.

**Supplementary Table 2**. Crystallographic data for **1**-$Br_{12}$.

| | **1**-$Br_{12}$ |
|---|---|
| **Chemical formula** | $C_{150}H_{132}Br_{12}N_6O_{12}S_6$ |
| **Formula weight** | 3361.89 |
| **Space group** | *P–3* |
| ***a*** **(Å)** | 23.028 (4) |
| ***b*** **(Å)** | 23.028 (4) |
| ***c*** **(Å)** | 19.626 (3) |
| $\alpha$ **(deg)** | 90 |
| $\beta$ **(deg)** | 90 |
| $\gamma$ **(deg)** | 120 |
| $V$ **(Å$^3$)** | 9013 (3) |
| ***Z*** | 2 |
| **μ (mm$^{-1}$)** | 4.21 |
| **T (K)** | 100 |
| $R1^a$ **(**$wR2^b$**)** | 0.157 (0.476) |
| **Reflections** | 3155 |
| **Radiation type** | Cu $K_\alpha$ |

$^aR1 = [\Sigma w(F_o - F_c)^2/\Sigma wF_o^2]^{1/2}$; $^bwR2 = [\Sigma[w(F_o^2 - F_c^2)^2]/\Sigma w(F_o^2)^2]^{1/2}]$, $w = 1/[\sigma^2(F_o^2) + (aP)^2 + bP]$, where $P = [\max(F_o^2,0) + 2(F_c^2)]/3$

## 3. PDF Analysis

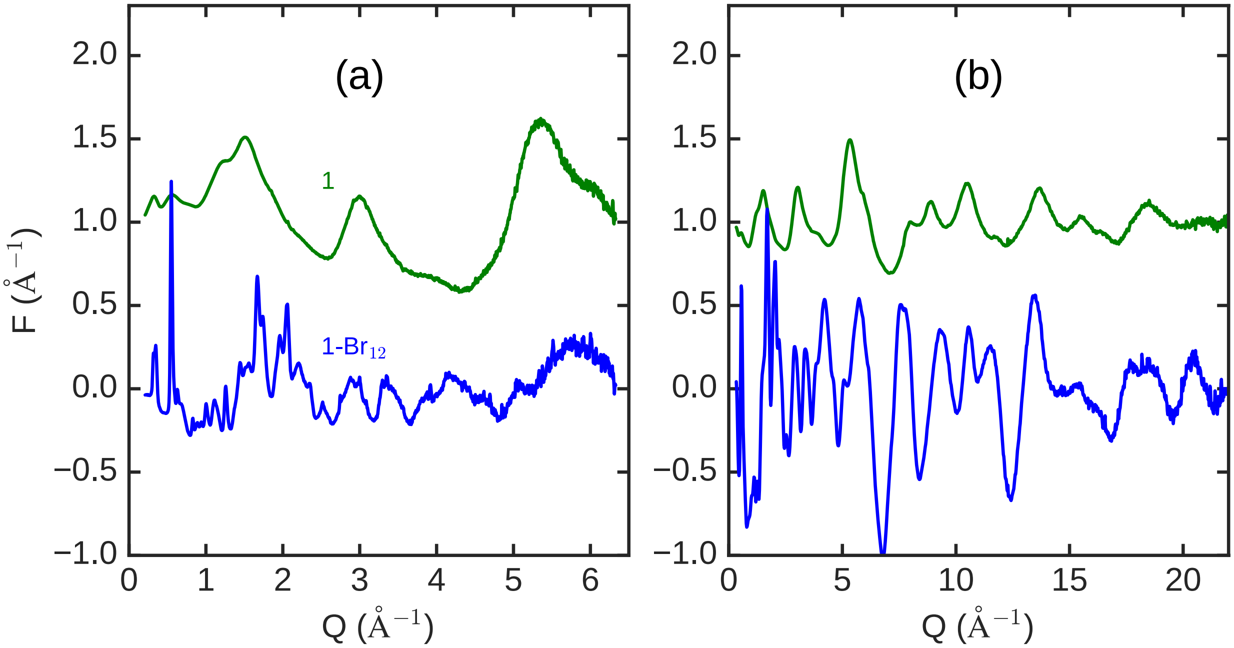


**Supplementary Figure 8**. The reduced scattering structure function *F(Q)* (given by *F(Q)* = *Q*[*S(Q)* – 1]) is shown for all four compounds in **a**, the far detector setup used for high *Q*-resolution, and **b**, the near detector setup used for measurement over a wide *Q*-range.

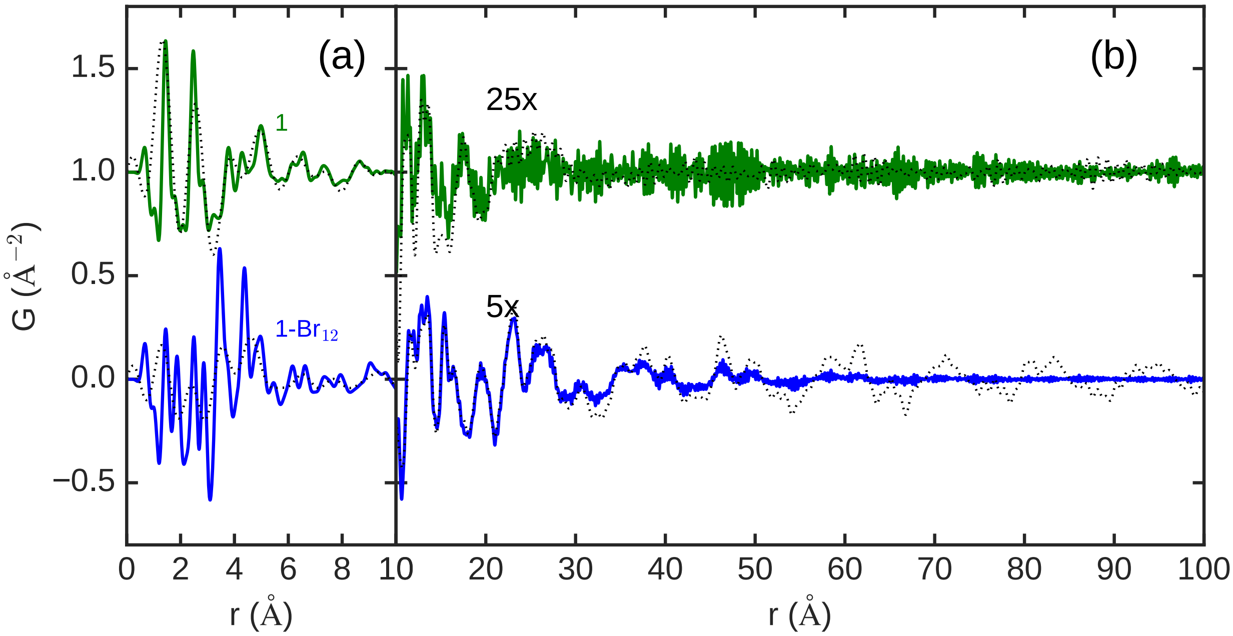


**Supplementary Figure 9. a,** The resulting PDFs, *G(r)*, are shown for all four compounds. PDFs from the near detector (high real space resolution) data are shown in color, and PDFs from the respective far detector measurements are shown as black dotted lines. **b,** Top and bottom data are magnified by 25x and 5x, respectively.

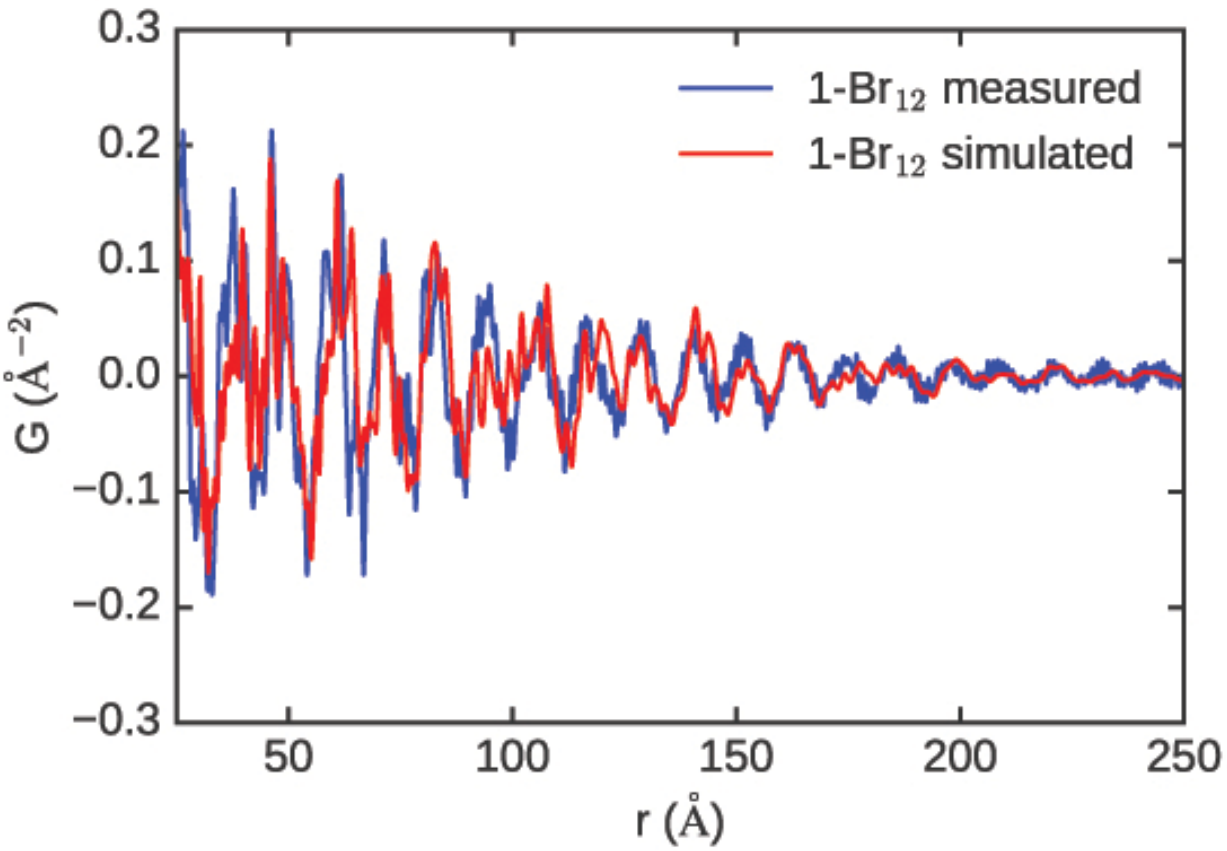


**Supplementary Figure 10**. The PDF for the brominated trimer (**1**-Br$_{12}$) from the far detector measurement is overlaid with a PDF simulated from the structure obtained from SCXRD. The simulated PDF was generated using the program PDFgui[16], calculated without any refinement, using experimental resolution parameters refined from the nickel standard data, ($Q_{damp}$ = 0.008 Å$^{-1}$ and $Q_{broad}$ = 0.015 Å$^{-1}$) the same $Q_{max}$ = 6.33 Å$^{-1}$, a global isotropic thermal displacement parameter, $U_{iso}$ = 0.03 Å$^2$, further damped by an envelope function with structural coherence of 400 Å, and rescaled for comparison. While there are differences, it is clear that the signals remain in phase over the whole range indicating that the long range molecular packing in the powdered sample is fairly well represented by that of the single crystal.

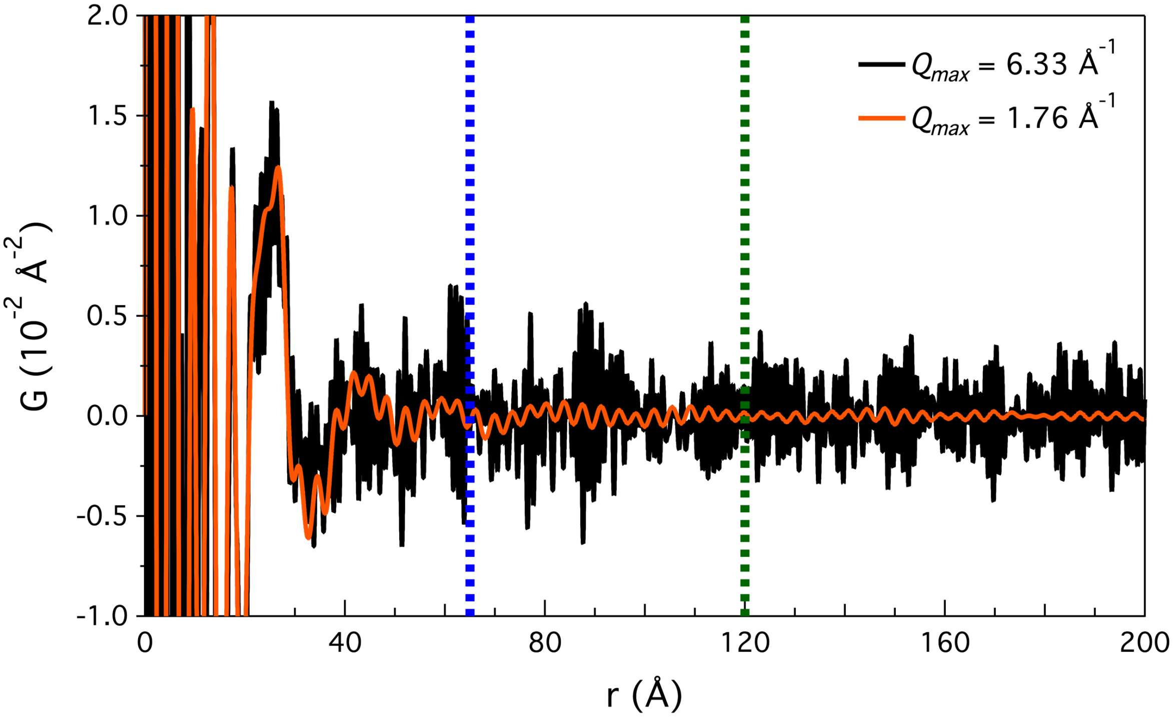


**Supplementary Figure 11**. Coherence length for **1**. The high $Q$-resolution, low $Q_{\max}$ measurement is shown in black ($Q_{\max}$ = 6.33 Å$^{-1}$). The high-$r$ signal is obscured by noise. We reduce noise by reducing further the $Q_{\max}$. Although reducing $Q_{\max}$ also reduces the real space resolution, the high-$r$ region is dominated by low frequency terms and therefore is still reliable for identifying structural signals. We therefore reduced the $Q_{\max}$ to 1.76 Å$^{-1}$ and visually locate where the signal becomes flat. We give an estimated lower bound on this value at 65 Å (blue dotted line) and upper bound of 120 Å (green dotted line).

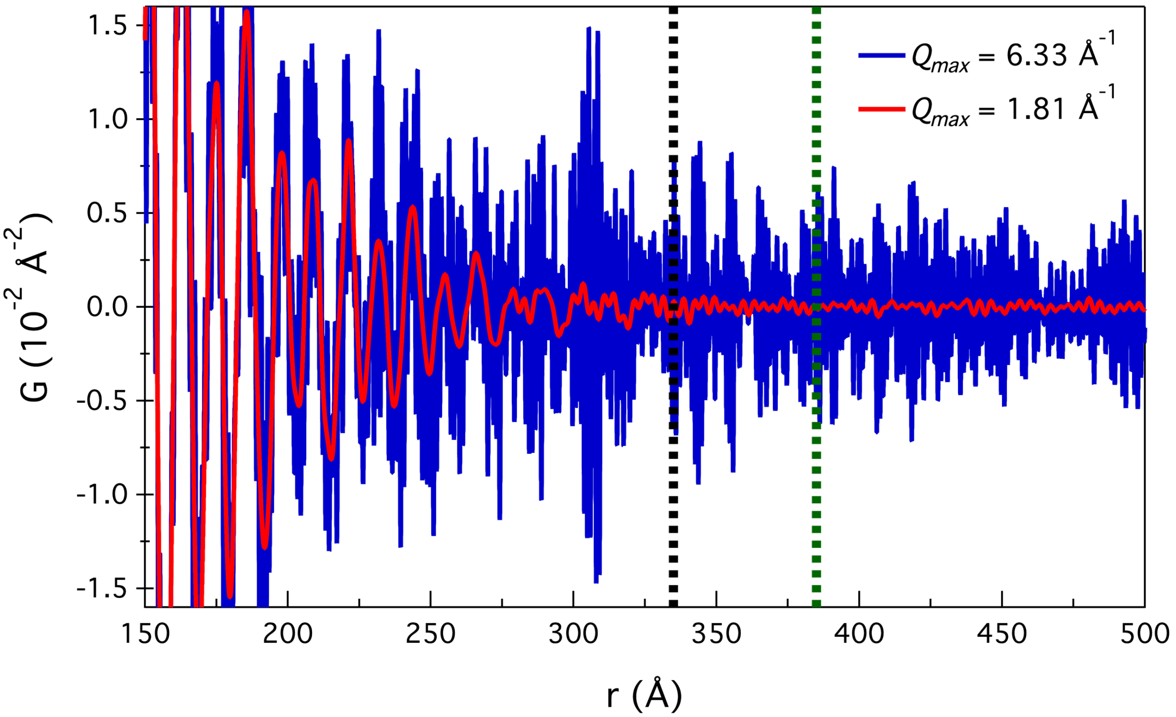


**Supplementary Figure 12**. Coherence length for **1**-$Br_{12}$. Following the same analysis described in Figure S11, we can approximate the coherence length lower bound at 335 Å (black dotted line) and upper bound at 385 Å (green dotted line).

## 4. Transfer and output curves of TFTs

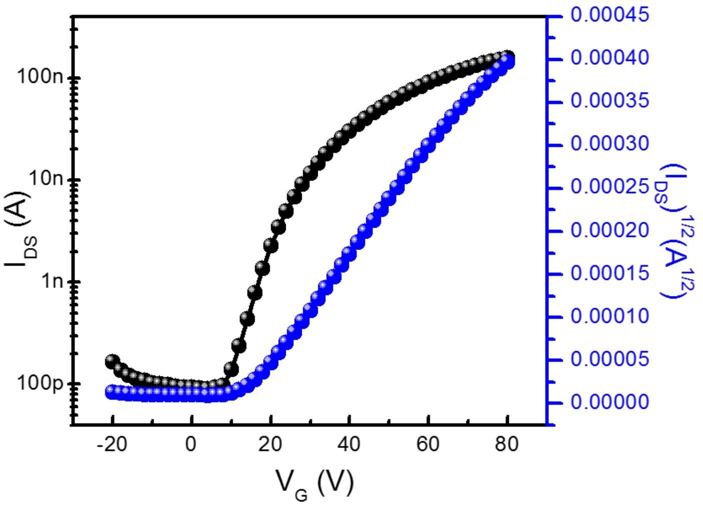


**Supplementary Figure 13.** Transfer characteristics of TFTs for **1**.

## 5. AFM height images of spin-cast films

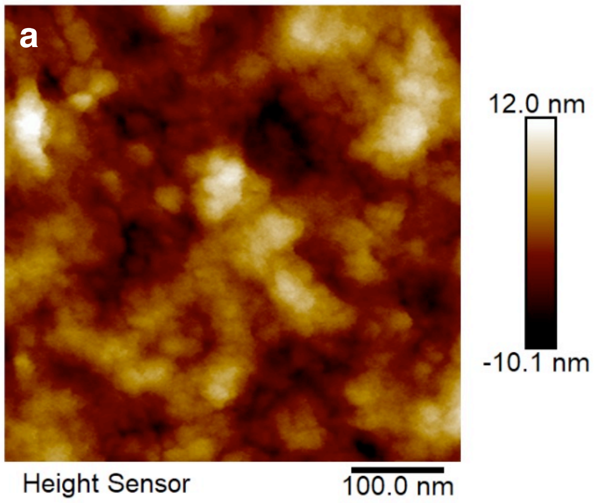


**Supplementary Figure 14.** AFM height images of spin-cast films for **a, 1**-$Br_{12}$ and **b, 1**. The root mean square roughness are 3.2 nm for **1**-$Br_{12}$ and 0.347 nm for **1**, respectively.

## 6. Measurement of exposure to gases

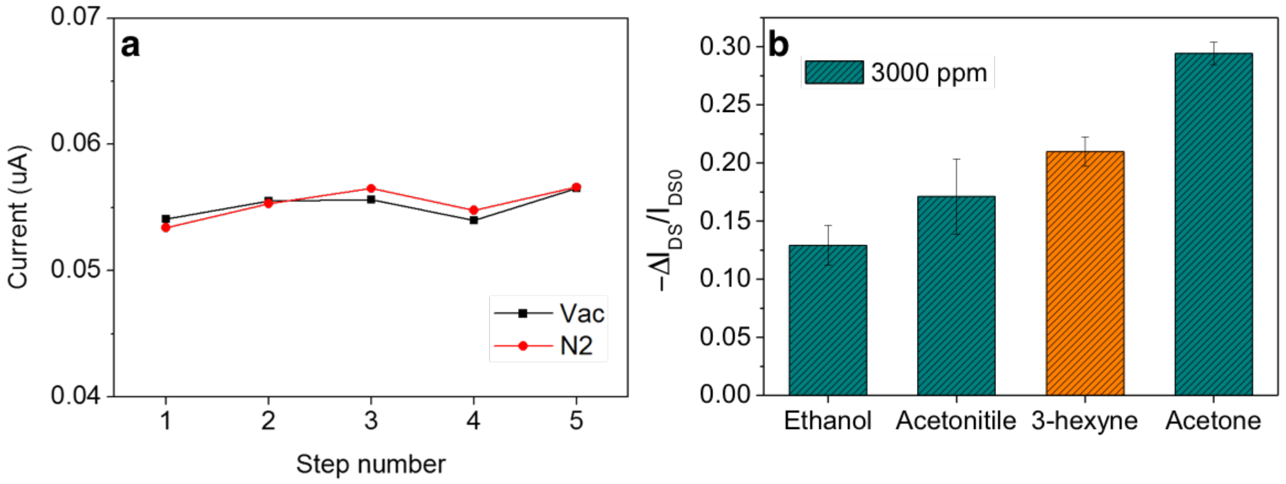


**Supplementary Figure 15. a,** Device cycling response for **1** under vacuum and $N_2$ atmosphere. **b**, The average current of response for a **1**-$Br_{12}$ TFT to series of analytes at a concentration of 3000 PPM. Error bars represent the standard error obtained in three measurements.

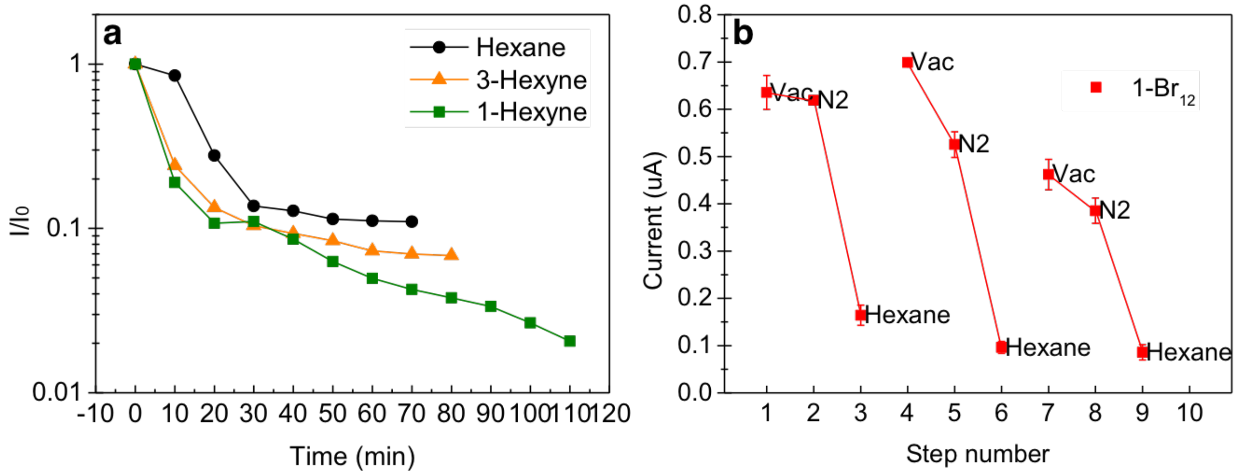


**Supplementary Figure 16. a**, $I/I_0$ for **1**-$Br_{12}$ under n-hexane, 3-hexyne and 1-hexyne. **b**, Device cycling response for **1**-$Br_{12}$ under vacuum, $N_2$ and hexane atmosphere. Error bars represent the standard error obtained in three measurements.

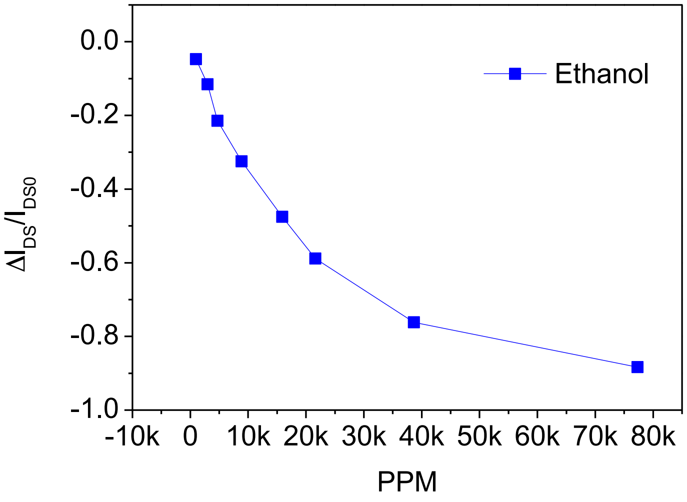


**Supplementary Figure 17.** Sensitivity for a **1**-$Br_{12}$ TFT under different concentration of ethanol.

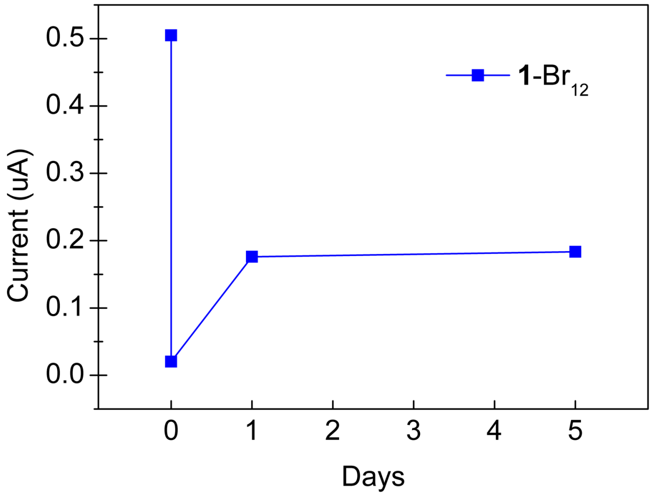


**Supplementary Figure 18.** Recovery response for **1**-$Br_{12}$ under vacuum after exposure to 1-hexyne.

## 7. $^{1}$H and $^{13}$C NMR spectra

**Stannane 2**

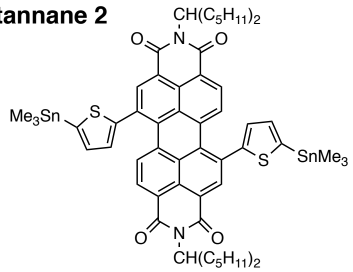


$^{1}$H NMR (500 MHz, $CDCl_3$)

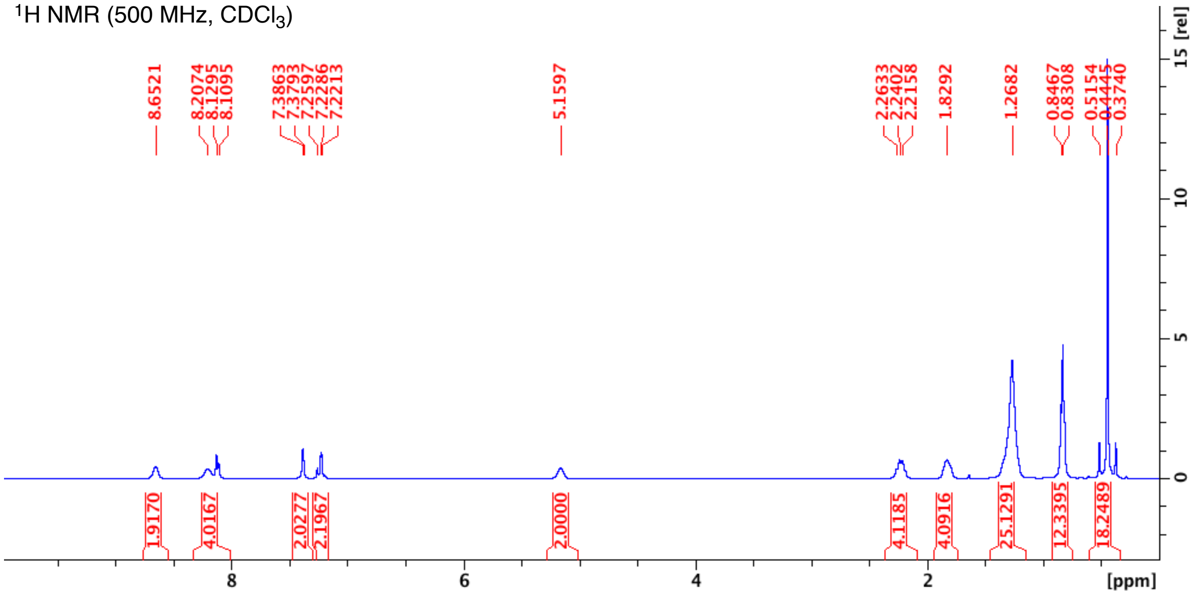


$^{13}$C NMR (100 MHz, $CDCl_3$)

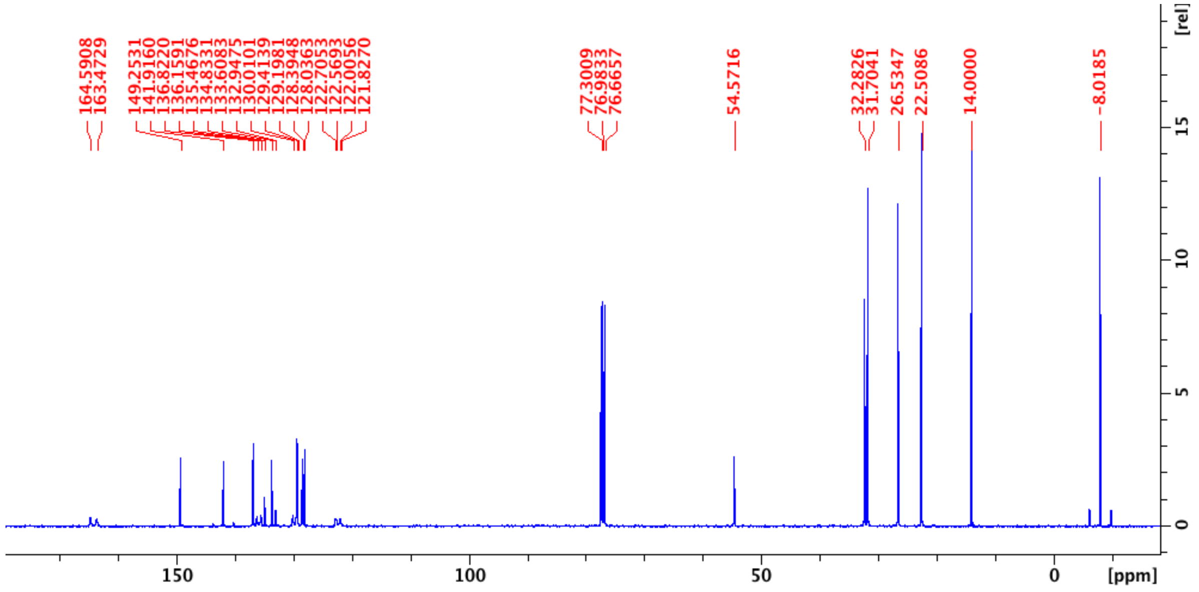

**Trimer 1**

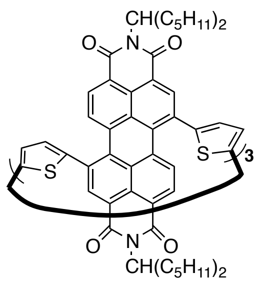


**$^1H$ NMR (500 MHz, $CD_2Cl_2$)**

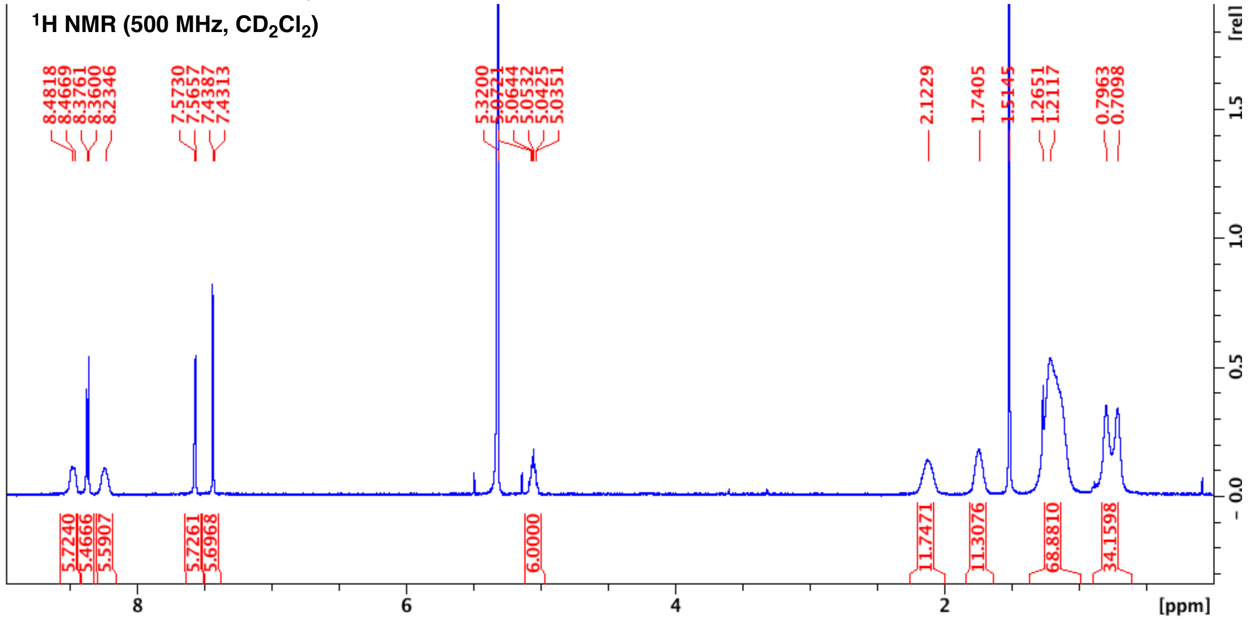


**COSY (400 MHz, $CDCl_3$)**

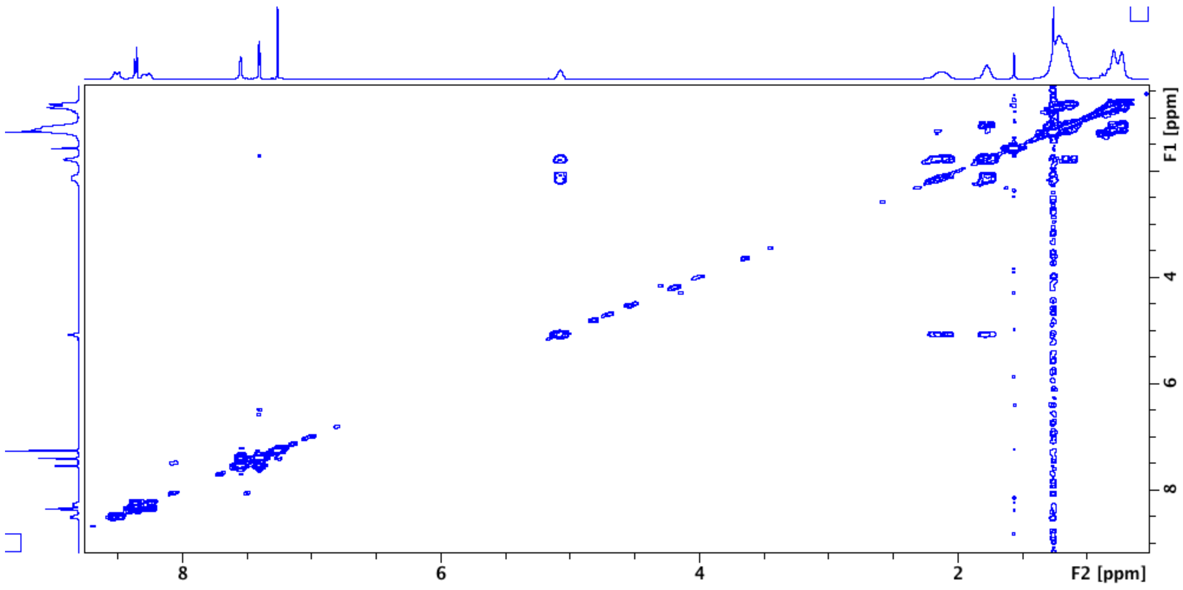

$^{13}C$ NMR (100 MHz, $CDCl_3$) **Trimer 1**

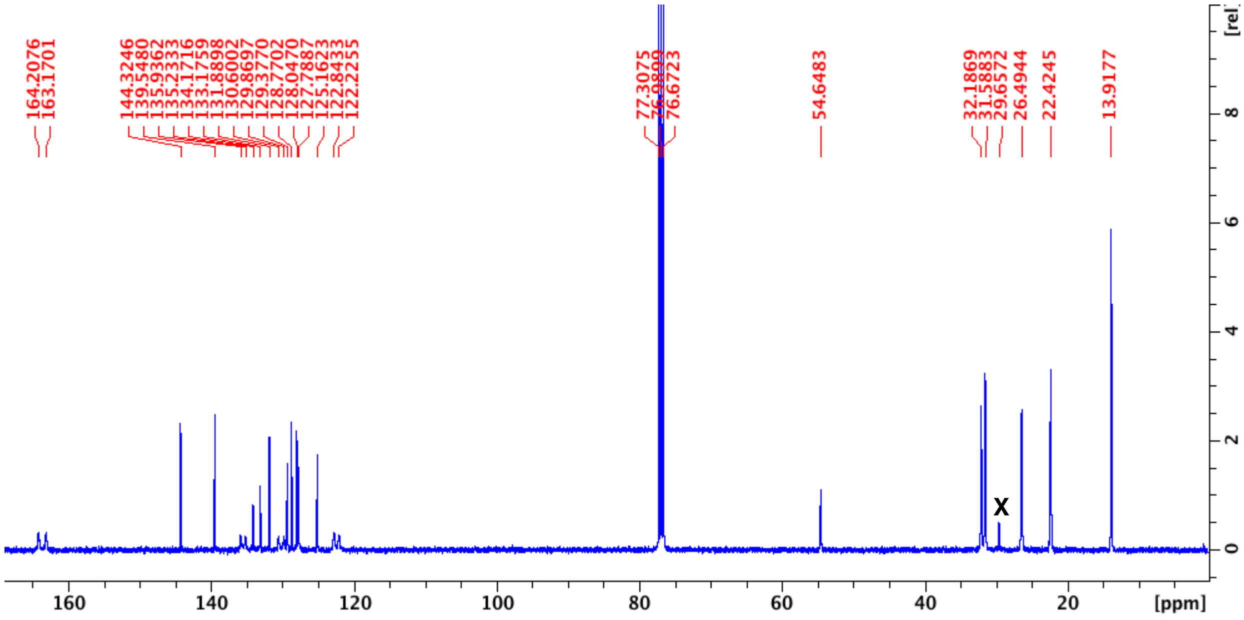


Chiral HPLC trace of trimer 1

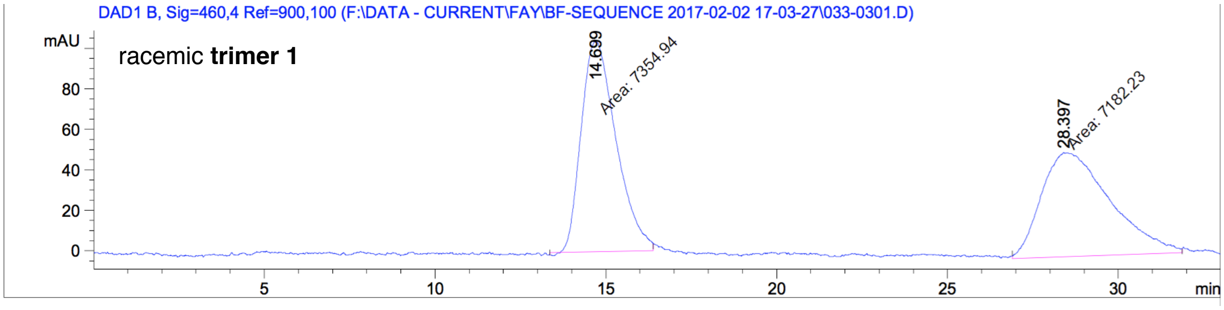


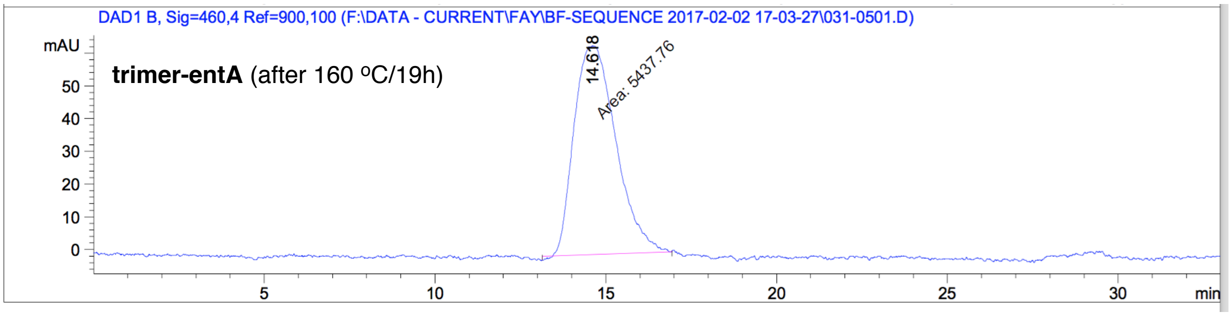


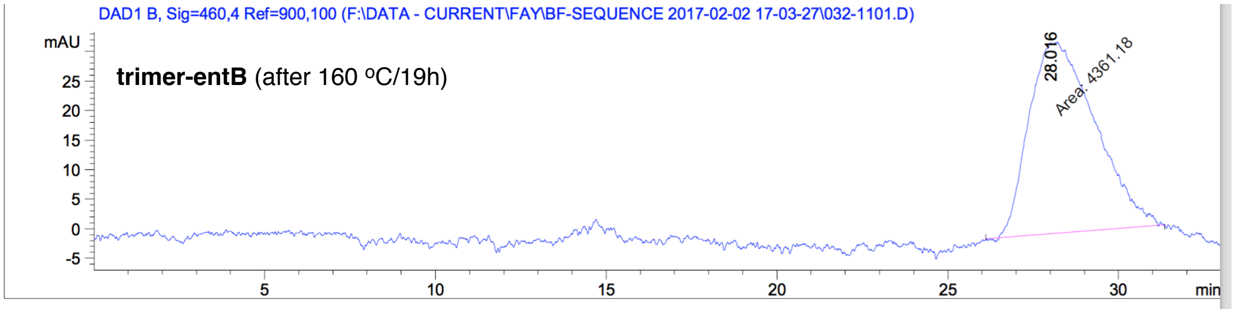

**Tetramer**

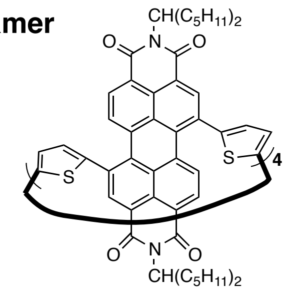


$^{1}H$ NMR (500 MHz, $C_2D_2Cl_4$, 333K)

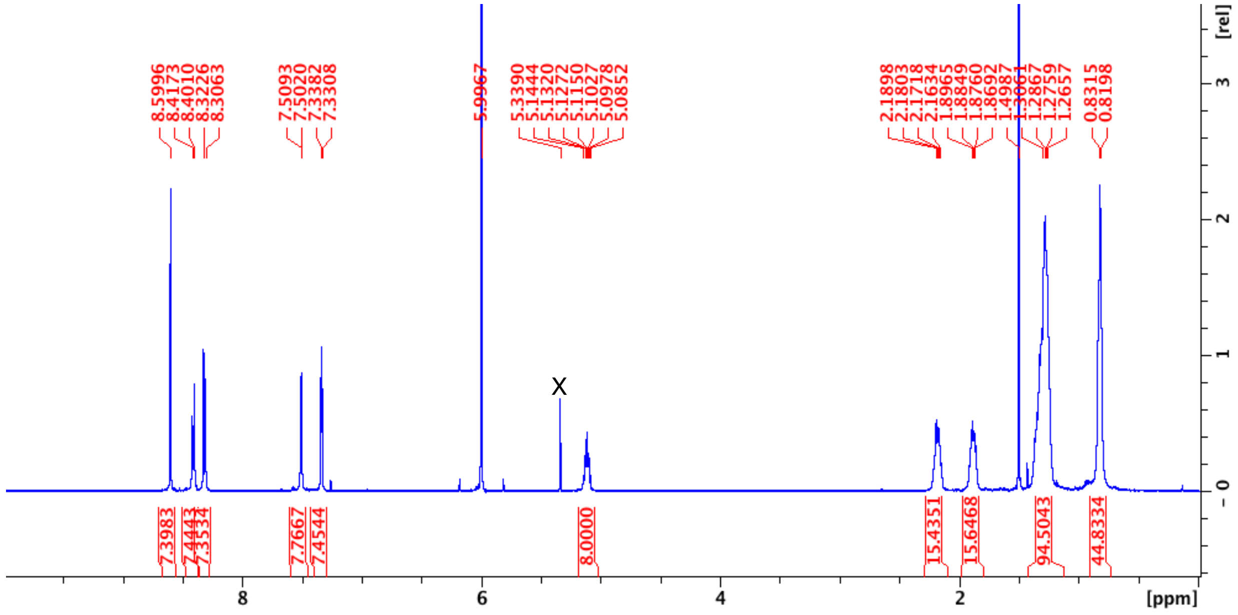


$^{13}C$ NMR (100 MHz, $CDCl_3$)

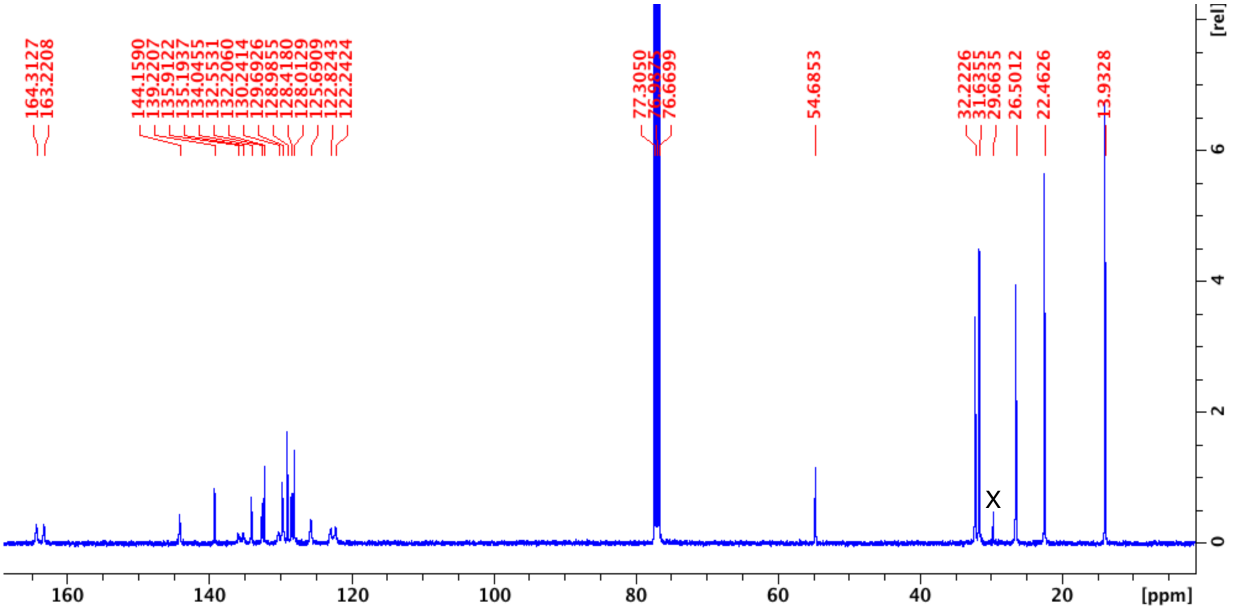

**Pentamer**

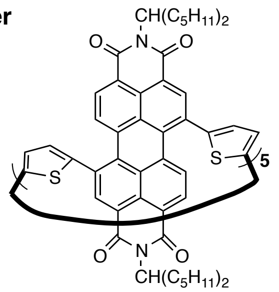


$^1H$ NMR (500 MHz, $CD_2Cl_2$)

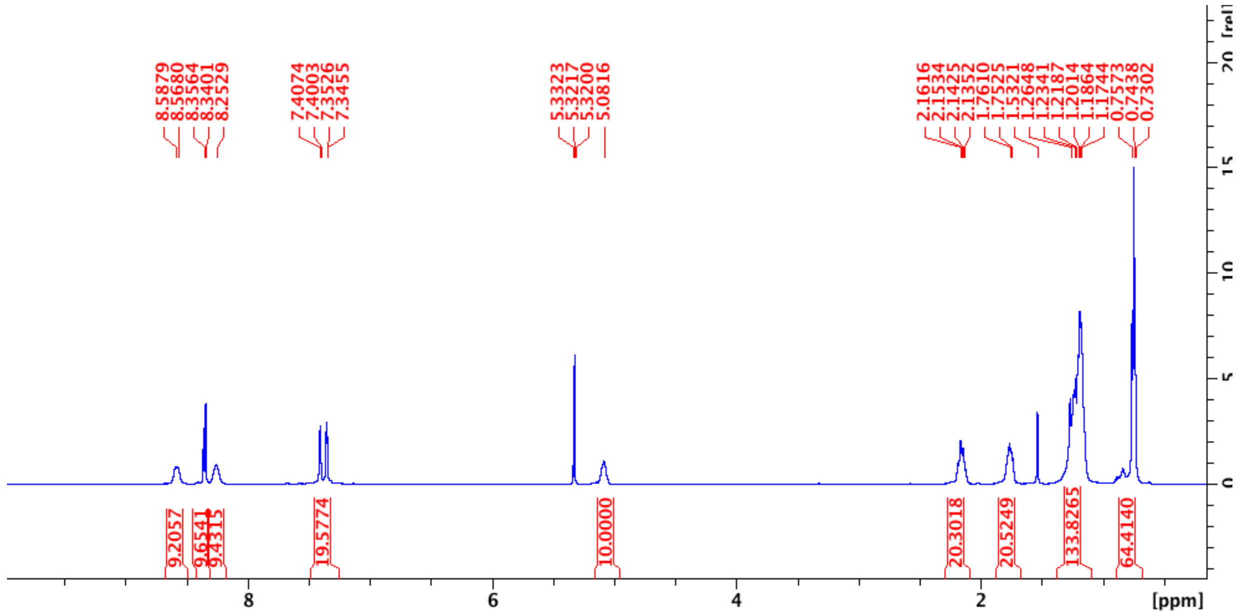

$^{13}C$ NMR (100 MHz, $CDCl_3$)

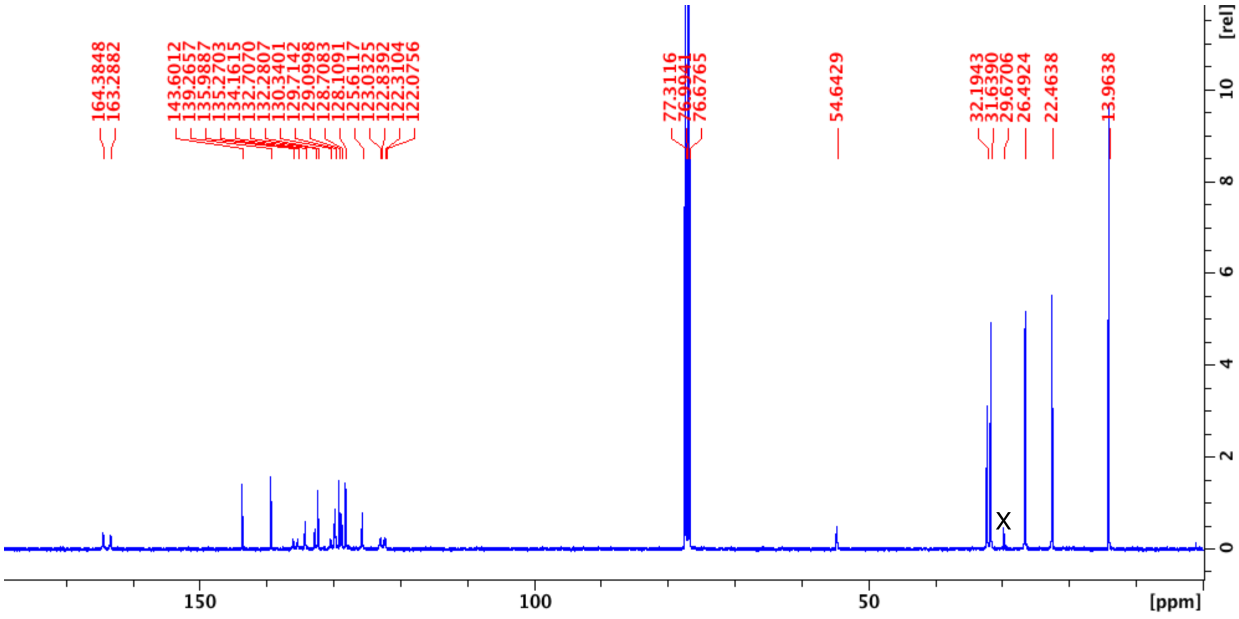

**Hexamer**

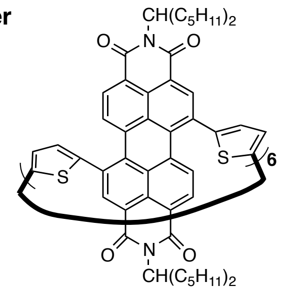


$^{1}H$ NMR (500 MHz, $CDCl_3$)

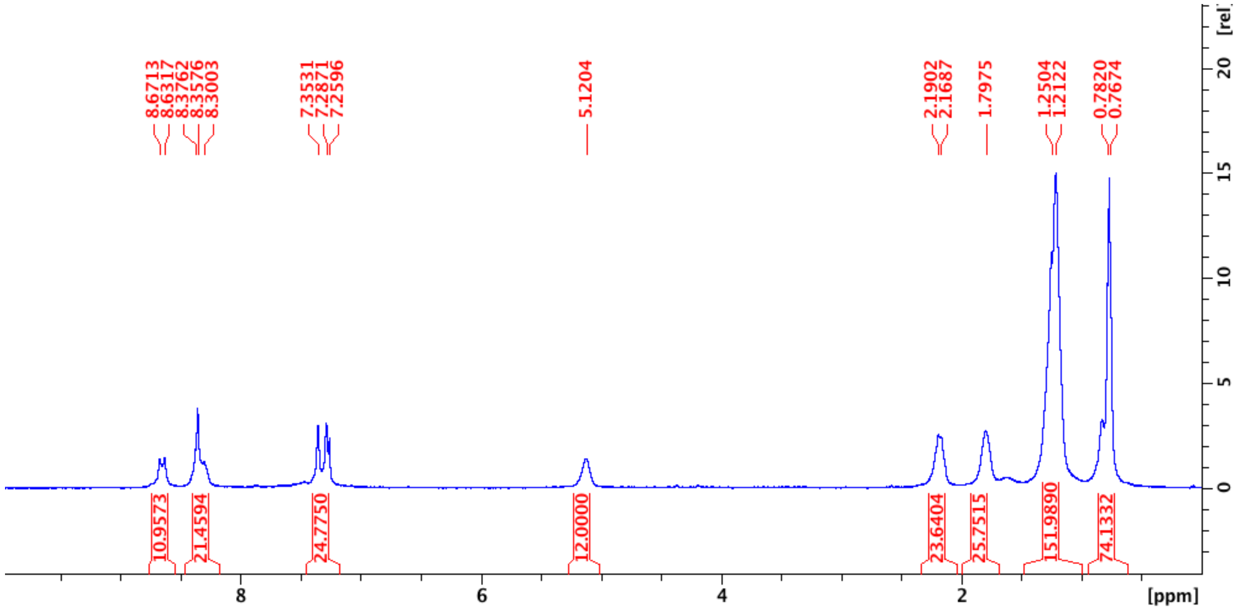


$^{13}C$ NMR (100 MHz, $CDCl_3$)

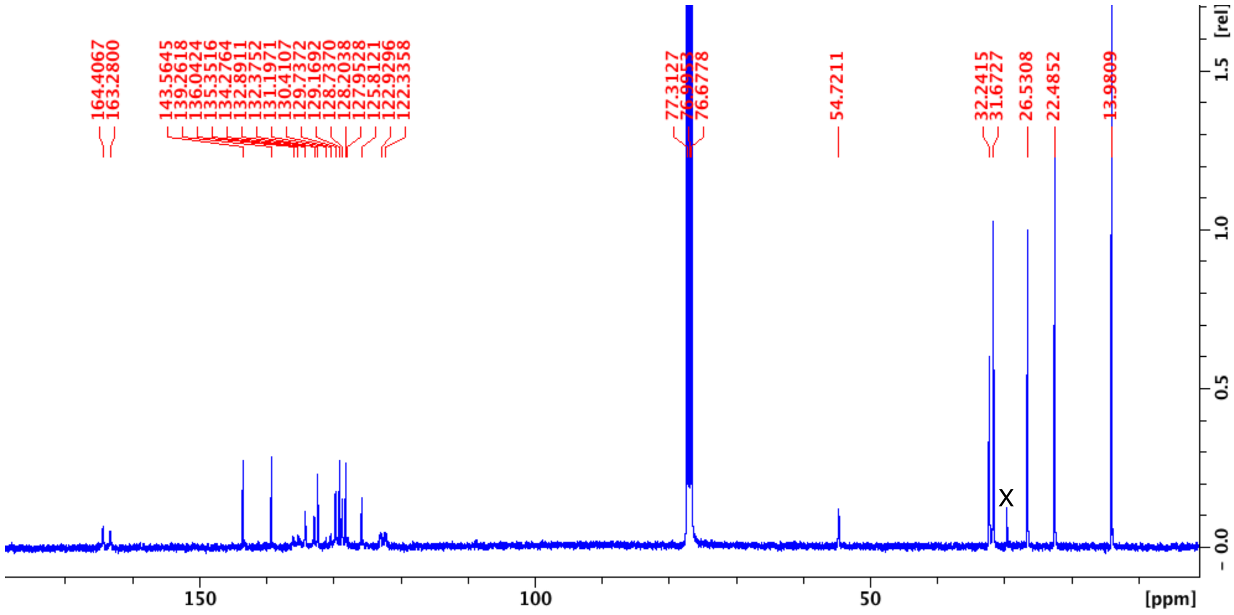

**Trimer 1-Br$_{12}$**

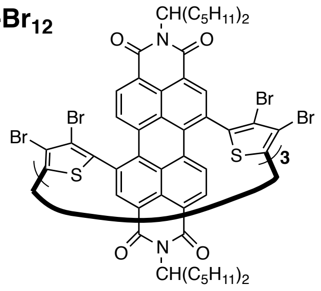


**$^1$H NMR (400 MHz, $CDCl_3$)**

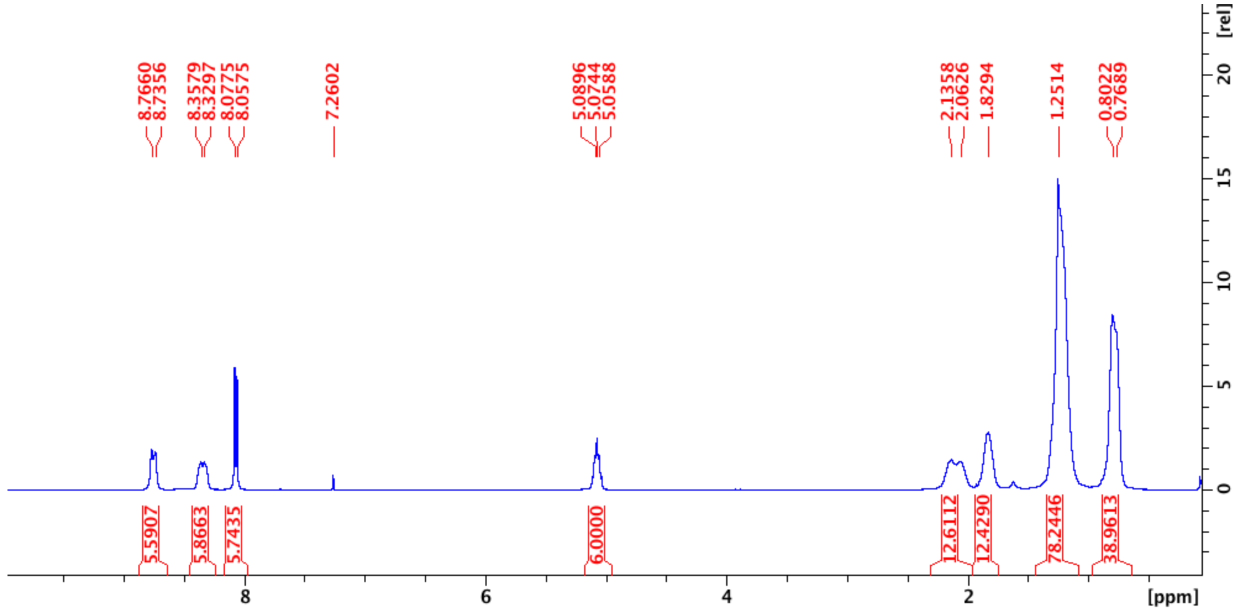


**$^{13}$C NMR (100 MHz, $CDCl_3$)**

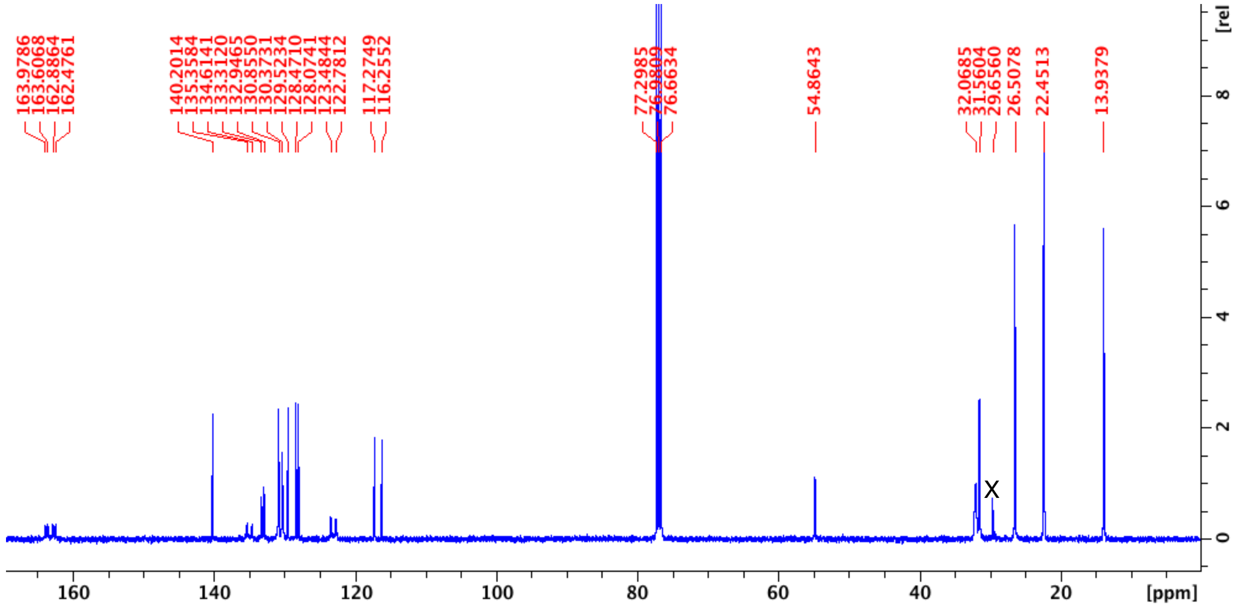

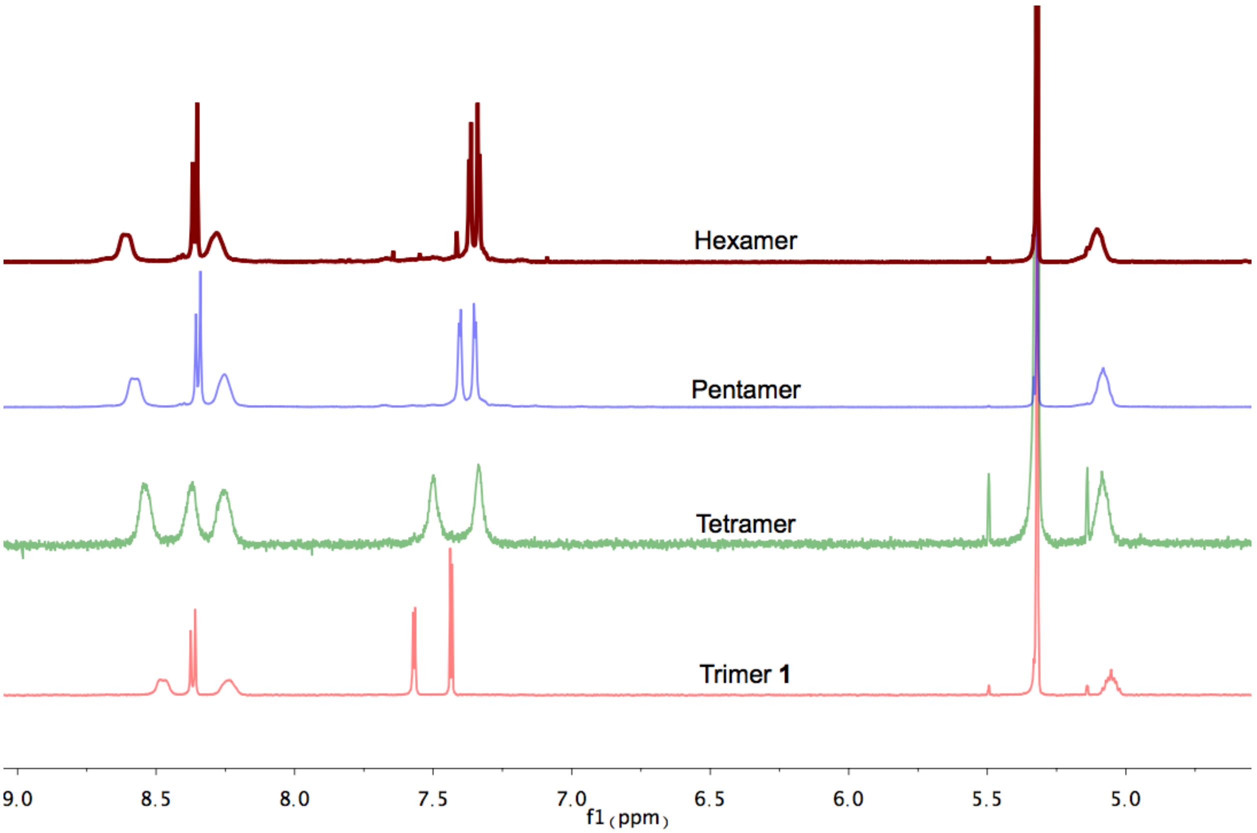


**Supplementary Figure 19.** Overlay of trimer to hexamer (500 MHz, $CD_2Cl_2$, 300K).

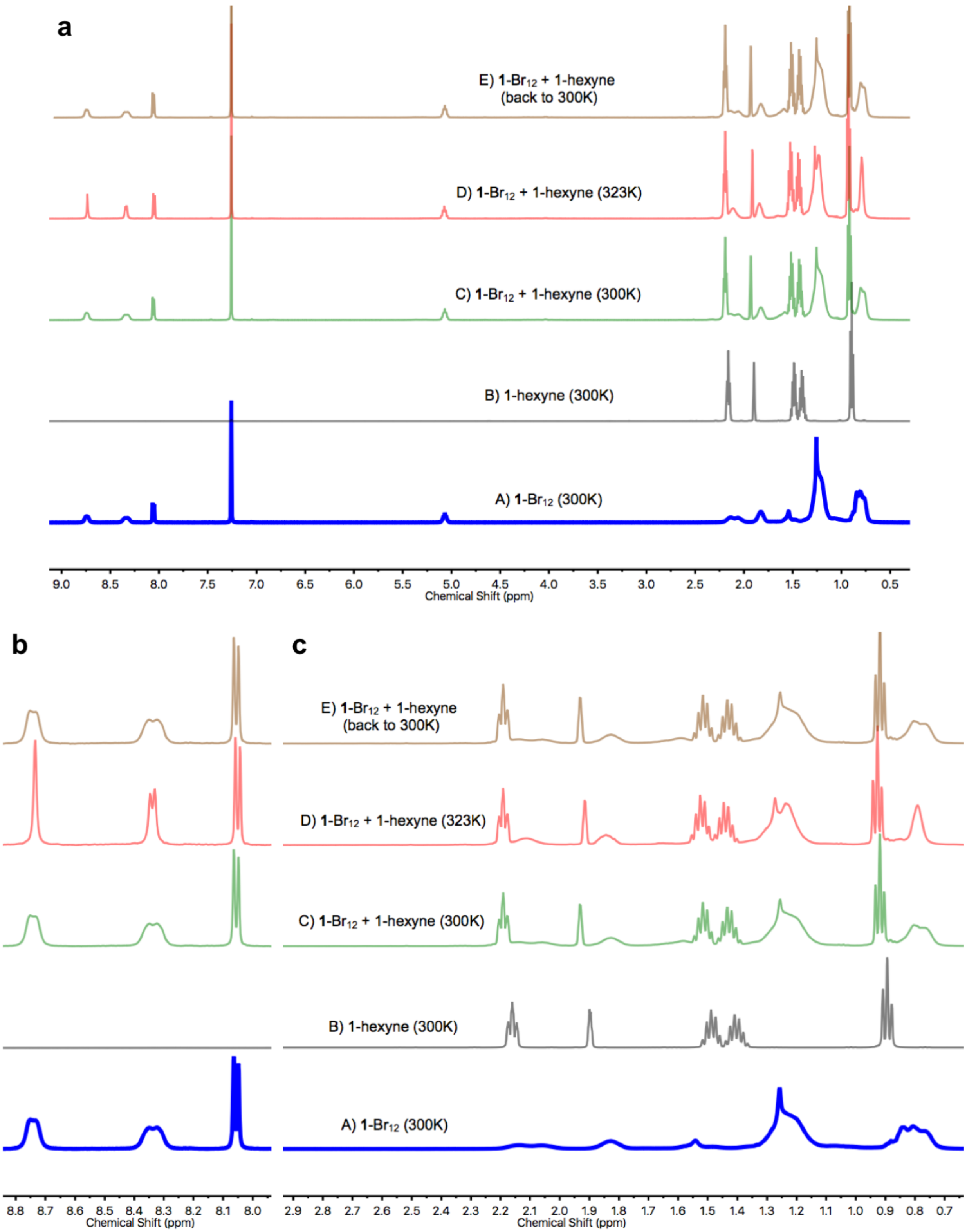


**Supplementary Figure 20.** Evidence for the lack of interaction or reaction between **1**-$Br_{12}$ and 1-hexyne in solution (500 MHz, $CDCl_3$). **a**, full spectra. **b** and **c**, zoomed-in plots.

## 8. Synthesis

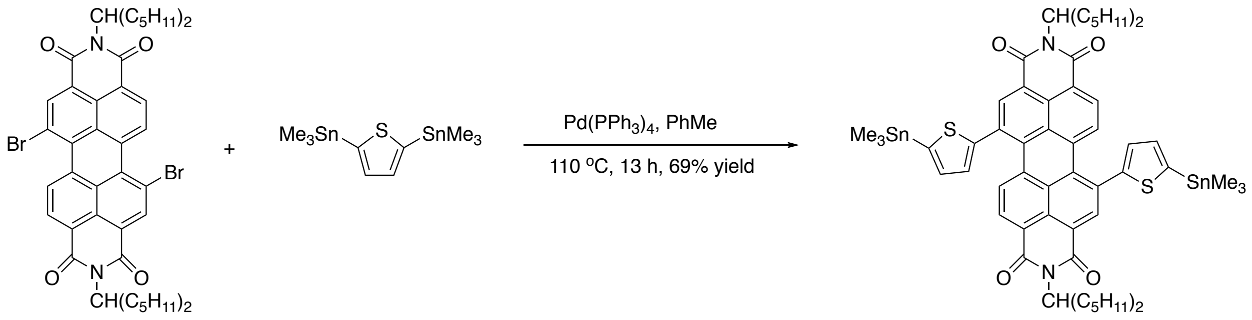


2

**Supplementary Figure 21.** Synthesis of stannane **2**.

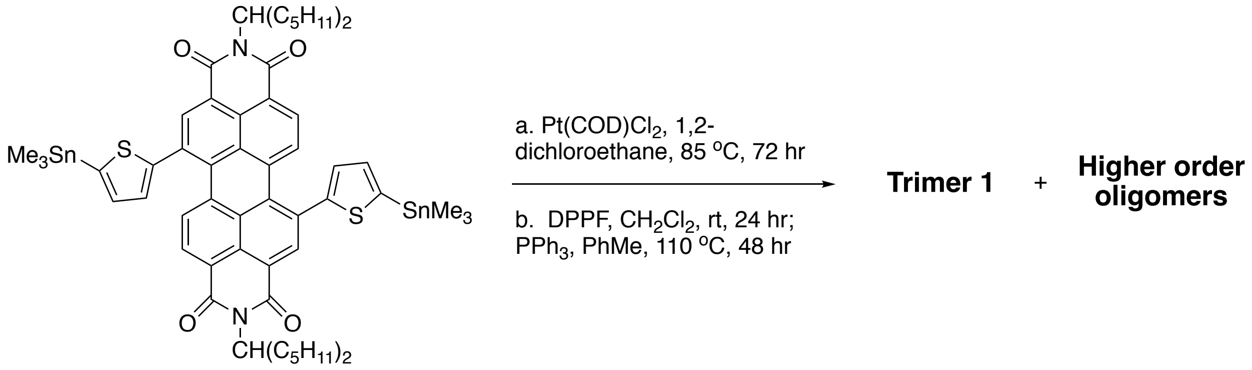


2

**Supplementary Figure 22.** Synthesis of Trimer **1**.

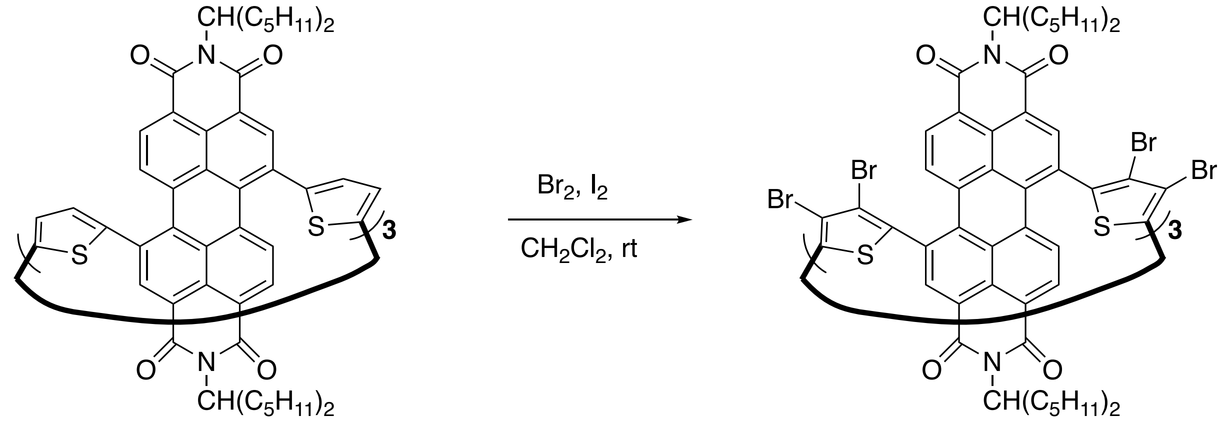


Trimer **1** Trimer **1**-$Br_{12}$

**Supplementary Figure 23.** Synthesis of **1**-$Br_{12}$.

## 9. Supplementary Methods

All reactions were performed in oven-dried or flame-dried round bottom flasks, unless otherwise noted. The flasks were fitted with Teflon magnetic stir bar, rubber septa and reactions were conducted under a positive pressure of nitrogen, unless otherwise noted. Anhydrous and anaerobic solvents were obtained from Schlenk manifold with purification columns packed with activated alumina and supported copper catalyst (Glass Contour, Irvine, CA). Automated flash chromatography was performed using a Teledyne Isco Combiflash $R_f$200 and Redisep $R_f$ Silica columns. Preparative high performance liquid chromatography (HPLC) purification was performed on a Waters Prep150 instrument equipped with a UV-vis detector, an automated fraction collector with either a Nacalai Tesque COSMOSIL Buckyprep column (20 mm ID x 250 mm, 5 μm) or a COSMOSIL 5PBB column (20 mm ID x 250 mm, 5 μm).

Chiral Analyses and Purification. Racemic trimer **1** samples were resolved by an Agilent 1200 Series analytical HPLC equipped with a diode array detector (300 nm to 900 nm) and a CHIRALPAK IA-3 column (4.6 mm ID x 250 mm, 3μm) from Chiral Technologies; preparative purification was performed using CHIRALPAK IA-3 column (21 mm ID x 250 mm, 5μm) on Waters Prep150 instrument.

***Materials***. Pure regioisomeric 1,7-dibromoperylene-3,4,9,10-tetracarboxylicbisimides was synthesized (as a mixture of 1,7- and 1,6-regioisomers) according to known procedures,[1] and successful separated using COSMOSIL Buckyprep column (20 mm ID x 250 mm, 5 μm) on Waters Prep150 instrument. All chemicals were purchased from commercial sources and used without further purification unless otherwise specified.

***Instrumentation***. $^{1}$H and $^{13}$C NMR spectra were recorded on Bruker DRX400 (400 MHz) or a Bruker DMX500 (500 MHz) spectrometer. Chemical shifts for protons are reported in parts per million downfield from tetramethylsilane and are referenced to residual protium in the NMR solvent ($CHCl_3$: δ 7.26; $CH_2Cl_2$: δ 5.32; $C_2H_2Cl_4$: δ 6.00). Chemical shifts for carbon are reported in parts per million downfield from tetramethylsilane and are referenced to the carbon resonances of the solvent ($CDCl_3$ δ 77.0; $C_2H_2Cl_4$ δ 73.78). Data are represented as follows: chemical shift, multiplicity (s = singlet, d = doublet, t = triplet, m = multiplet, br = broad), coupling constants in Hertz, and integration. Some $^{1}$H NMR and $^{13}$C NMR were recorded at elevated temperatures (in K) to enhance peak resolution in the aromatic region. Resonances corresponding to the numerous

aromatic carbon atoms in the reported compound sometimes overlap, thereby reducing the number of observed resonances.

High-resolution mass spectrometry (HRMS) was performed on (1) a Waters XEVO G2-XS QTOF instrument equipped with a UPC2 SFC inlet, and electrospray (ESI) and atmospheric pressure chemical (APCI) ionization sources; or (2) a Bruker UltrafleXtreme MALDI TOF instrument using dithranol matrix.

Absorption spectra were obtained on Shimadzu UV 1800 UV-Vis spectrophotometer and emission spectra were recorded in a Fluorolog-3 spectrophotometer.

Cyclic voltammograms (CVs) were recorded on a CHI600C electrochemical workstation using Ag/AgCl electrode as the reference electrode. 0.1 M solution tetrabutylammonium hexafluorophosphate, $[Bu_4N][PF_6]$, in dichloromethane was used as the supporting electrolyte. The thin film transistors were tested on the Agilent 4155C semiconductor parameter analyzer. CD spectra were recorded by a Jasco J-810 spectropolarimeter. It is assumed that the absolute energy level for $Fc/Fc^+$ redox potential is −4.80 eV with respect to vacuum level. The energy levels of the lowest unoccupied molecular orbitals (LUMO) and highest unoccupied molecular orbitals (LUMO) are calculated according to the following equation: $E_{LUMO} = -e(E_{red}+4.80)$ (eV), $E_{HOMO} = -e(E_{oxi}+4.80)$ (eV).

Atomic force microscopy (AFM) was performed with a PSIA XE100.

Powder X-ray diffraction data were collected on a PANalytical X'Pert[3] Powder Diffractometer. Data was collected on powder samples and films drop-cast from chloroform solution and p-xylene solution. For all data collection a Si zero-background holder was used. Single crystal data for **1**-$Br_{12}$ was collected on an Agilent SuperNova diffractometer using a mirror-monochromated Cu $K_\alpha$ radiation. The hexagonal-shaped crystals were mounted on a MiTeGen Kapton loop (polyimide). These were cooled to 100 K with an Oxford Cryosystems nitrogen flow apparatus. Data integration, scaling (ABSPACK) and absorption correction were perfomed in CrysAlisPro.[2] Structure solution was performed using ShelXS,[3] ShelXT,[4] or SuperFlip.[5] Subsequent refinement was performed by full-matrix least-squares on $F^2$ in ShelXL. Olex2[6] was used for viewing and to prepare CIF files. PLATON[7] was used for SQUEEZE,[8] ADDSYM[9] and TwinRotMat. Details of crystallographic data and refinement parameters are given in Table S2. Due to heavy disorder of the alkyl imide chains in **1**-$Br_{12}$, only nine carbons (out of eleven) in each alkyl fragment were modeled. The cavity size of **1**-$Br_{12}$ was calculated from the

solvent accessible volume calculator in Olex2. By employing this functionality we found discrete pockets within the structure of **1**-$Br_{12}$ which match the cavities of these molecules. Thus, the calculated cavity size of (SSS/RRR)-**1**-$Br_{12}$ is 414.9 Å$^3$ (CalcSolv 3.0 Å probe, grid step 0.2 Å).

X-ray total scattering experiments (PDF analysis) were conducted on beamline 28-ID-2 at the National Synchrotron Light Source II at Brookhaven National Laboratory. An X-ray beam of energy 67.756 keV (λ = 0.18299 Å) was focused on samples loaded into Kapton capillaries. Scattered intensities were collected at room temperature, in rapid acquisition mode[10] on a Perkin-Elmer 2D flat panel detector (2048 x 2048 pixels and 200 x 200 μm pixel size) mounted orthogonal to the beam path. Data collection was carried out with a short (239.9734 mm) and a large (1552.689 mm) sample-to-detector distance to obtain improved $Q$-space resolution. $Q$ is the magnitude of the scattering momentum transfer where for elastic scattering is defined as: $Q = 4\pi \sin(\theta)/\lambda$, for scattering angle 2θ and wavelength λ. A Ni standard sample was measured in both cases to calibrate the detector geometry. 2D intensities were azimuthally integrated to 1D intensities versus $Q$ using Fit2D[11]. Scattering from an empty Kapton tube was measured for background subtraction. The pair distribution function (PDF) gives the scaled probability of finding atom-pairs in the material at a distance $r$ apart. The program xPDFsuite with PDFGetX3[12, 13] was used to obtain the PDFs from the experimental scattering intensities. The coherent scattering $I(Q)$ was extracted through background subtraction and corrections to the raw intensities, then normalized by the atomic scattering factors to give the total scattering structure function $S(Q)$ which is converted to the real-space pair distribution function (PDF), $G(r)$, by

$$G(r) = \frac{2}{\pi}\int_{Q_{\min}}^{Q_{\max}} Q[S(Q) - 1](Q)\sin(Qr)\,dQ$$

where $Q_{min}$ and $Q_{max}$ are the minimum and maximum values of the scattering momentum transfer considered. These limits were determined by the $Q$-range accessible for the different detector configurations, 0.29–22.0 Å$^{-1}$ for the short sample-to-detector distance which gives a wide range of momentum transfer in order to achieve a high real-space resolution in the PDFs. The far sample-to-detector distance gives a smaller $Q$-range of 0.20–6.33 Å$^{-1}$, which gives a lower real space resolution, but provides a much better $Q$-resolution of the scattering which allows the resulting PDFs to be analyzed over longer real-space distances[14, 15] In this case, the $Q_{max}$ was reduced further

to decrease the noise level below any signal observable at high distances. The structural coherence of the sample was estimated from visual observation of the distance at which the structural signal became indistinguishable from the average atomic density, $G(r) = 0$.

It is important to note the following for the coherence length estimation: (1) all approximated coherence lengths are well below the resolution limit determined from fitting Ni measured with the same experimental setup, and (2) the approximated lengths may be underestimated as the presence of noise may still obscure some high-$r$ signals.

***Electron Transport.*** We first treat the substrate (300 nm of $SiO_2$ on a Si wafer) with octadecyltrichlorosilane (OTS) in order to passivate traps on the $SiO_2$ surface. Au source and drain electrodes are deposited on the film to make a bottom-contact configuration. We then spin-cast films of **1** and **1**-$Br_{12}$ onto this surface at 1000 r.p.m. for 1 min, to form transistors using the silicon wafer as the global back gate for the device. The thickness of the organic films is 15−20 nm. Thin Film Transistors (TFTs) made from thicker films (40−60 nm) exhibit nonlinear characteristic at low bias voltage. Finally, the film of **1**-$Br_{12}$ was annealed under p-xylene vapor for 10 min and then annealed under inert atmosphere at 160 °C for 10 mins to optimize the device performance. The film of **1** was annealed under inert atmosphere at 200 °C for 10 mins to optimize the device performance. Vapor annealing of p-xylene didn't show enhanced performance for **1**.

The mobility is calculated in the saturation regime using $I_{DS} = (W/2L)C_i\mu(V_G - V_T)^2$, where $W$ and $L$ are the width and length of the channel, $C_i$ (11.5 $nFcm^{-2}$), $\mu$, and $V_T$ correspond to the capacitance per unit area of the gate insulator, the field effect mobility, and the threshold voltage, respectively. $W$ = 105 μm and $L$ = 20 μm for transistor devices. $W$ = 2 mm and $L$ = 10 μm for sensor devices.

The crystalline material **1-**$Br_{12}$ provide a unique opportunity to study how thin film assembly and charge transport are effected by the dynamics of the semiconducting subunits. This is important because, if films can be made to maintain the open spaces on the interior of the hollow semiconductor, they could be used as a locus for guest incorporation to modulate the semiconducting properties. The crystalline material made here, **1-**$Br_{12}$, possesses an enormous amount of space within their interiors.

A saturated vapor of the analyte or odor is delivered to the thin film transistor using a bubbler. Nitrogen was used as the carrier gas. A sealed exposure chamber with a volume of 1.7 L was used; the flow rate of $N_2$ through the chamber was 0.2 $Lmin^{-1}$. Responsing experiments were

conducted by fixing the drain voltage at the determined minimum saturation value and conducting gate sweeps. The gate was applied in a pulsed manner in order to reduce device hysteresis. The responses of these devices to analytes were investigated by plotting the percent change in drain current, $I_{DS}/I_{DS,0}$ (measured at $V_G$ = 80 V, $V_{DS}$ = 80 V) versus time of exposure to analytes. For concentration dependent measurement, the analyte concentration was set via a saturated vapor of the analyte or odor using a bubbler, subsequently diluted through a series of gas mass-flow controllers (FMA-5506A and FMA-5514A). During the course of experiment, the total fow rate of the $N_2$ gas was set to a constant 500 standard cubic centimeter (SCCM).

***Synthesis details.*** Synthesis of stannane **2** (Supplementary Figure 21): A solution of 1,7-dibromoperylene-3,4,9,10-tetracarboxylicbisimides (1.145 g, 1.34 mmol, 1.00 equiv) in toluene (15 mL) was degassed under nitrogen for 20 minutes. In a separate flask, a solution of 2,5-bis(trimethylstannyl)thiophene (2.64g, 6.44 mmol, 4.81 equiv) in toluene (20 mL) was degassed under nitrogen for 20 minutes. The 2,5-bis(trimethylstannyl)thiophene solution was transferred under nitrogen to the dibromoperylene solution, and degassed for 40 minutes. Tetrakis(triphenylphosphine)palladium(0) (70 mg, 0.06 mmol, 4 mol%) was added, and the resultant solution was degassed for 20 minutes. The mixture was placed in oil bath set at 115 $^{o}$C for 13 h under nitrogen. The resultant blue reaction mixture was concentrated via rotoevaporation under reduced pressure. The product was purified using C18 reverse phase column chromatography (DCM:acetonitrile 1:1) to yield blue-purple solid **2** (1.10 g, 0.93 mmol, 69%). **$^{1}$H NMR** (500 MHz, $CDCl_3$): $\delta$ 8.65 (br, 2H); 8.21 (br, 2H); 8.12 (d, J = 8 Hz, 2H); 7.38 (d, J = 3 Hz, 2H); 7.22 (d, J = 3Hz, 2H); 5.16 (m, 2H); 2.26-2.22 (m, 4H); 1.83 (m, 4H); 1.28 (m, 24H); 0.85 (m, 12H); 0.46*(s, 18H). **$^{13}$C NMR** (100 MHz, $CDCl_3$): $\delta$ 164.59; 163.47; 149.25; 141.92*; 136.82; 136.16; 135.47; 134.83; 133.61; 132.95; 130.01; 129.41; 129.20; 128.39; 128.04; 122.71; 122.57; 122.01; 121.83; 54.57; 32.28; 31.70; 26.53; 22.51; 14.00; -8.02*. **IR** (ATR-ZnSe) [cm$^{-1}$] 3025, 2966, 2929, 2909, 2842, 2723, 2244, 1628, 1427, 1371. **HRMS** (APCI+) calculated m/z for $[C_{60}H_{74}N_2O_4S_2Sn_2+H]^+$ 1189.3225; found 1189.3237.

*Tin satellite peaks visible.

Synthesis of Trimer **1** (Supplementary Figure 22): A solution of stannane **2** (833 mg, 0.70 mmol, 1.00 equiv) and dichloro(1,5-cyclooctadiene)platinum (262 mg, 0.70 mmol, 1.00 equiv) in 1,2-dichloroethane (180 mL, 3.9 mM) was placed in oil bath set at 85 $^{o}$C under $N_2$ for 3 days. The blue solution was concentrated to a dark blue solid that was washed with hexane. The resultant

solid was dried under vacuum. It was combined with 1,1′-ferrocenediyl-bis(diphenylphosphine) (550 mg, 1.00 mmol, 1.43 equiv) in $CH_2Cl_2$ (100 mL). The solution was set to stir under $N_2$ for 2 days. The solution was concentrated, and redissolved in toluene (100 mL) and combined with triphenylphosphine (2.01 g, 7.12 mmol, 10.17 equiv). The resultant solution was set to reflux under $N_2$ for 2 days. The reaction mixture was concentrated under reduced pressure, the resultant dark blue solid was subjected to Soxhlet extraction: hexane, methanol, acetone, and $CH_2Cl_2$. The $CH_2Cl_2$ extract contained the cyclic and acyclic compounds. The resultant solid was first purified by alumina chromatography using gradient of 50% $CH_2Cl_2$/hexane to 80% $CH_2Cl_2$/hexane. The resultant fractions with cyclic and acyclic compounds were purified into its individual cyclic components by HPLC with a 5PBB column eluting with 27% $CH_2Cl_2$/hexane. Trimer **1** (90 mg, 0.035 mmol, 15% yield); tetramer (62 mg, 0.018 mmol, 10% yield); pentamer (32 mg, 0.007 mmol, 5% yield); hexamer (26 mg, 0.005 mmol, 4% yield). The remaining mass balance is higher order cyclic and acyclic oligiomers.

Trimer **1**: **$^1$H NMR** (500 MHz, $CD_2Cl_2$): $\delta$ 8.48-8.47 (br, 6H); 8.37 (d, J = 8.1 Hz, 6H); 8.23 (br, 6H); 7.57 (d, J = 3.7 Hz, 6H); 7.43 (d, J = 3.7 Hz, 6H); 5.07-5.04 (m, 6H); 2.12 (br, 12H); 1.74 (br, 12H); 1.26-1.21 (m, 72H); 0.80-0.71 (m, 36H). **$^{13}$C NMR** (100 MHz, $CDCl_3$): $\delta$ 164.21; 163.17; 144.32; 139.55; 135.94; 135.23; 134.17; 133.18; 131.89; 130.60; 129.87; 129.38; 128.77; 128.05; 127.79; 125.16; 125.16; 122.84; 122.22; 54.65; 32.19; 31.59; 26.49; 22.42; 13.92. **IR** (ATR-ZnSe) [cm $^{-1}$] 2924.4; 2856.6; 1696.9; 1655.7; 1584.9; 1455.0; 1400.9; 1321.2; 1243.0; 1180.2; 1118.0; 974.9; 911.8; 859.2; 832.8; 810.1; 756.7; 713.5. **HRMS** (MALDI, dithranol matrix) calculated m/z for $[C_{162}H_{168}N_6O_{12}S_6]^-$ 2581.1039; found 2581.1036.

**Tetramer**: **$^1$H NMR** (500 MHz, $C_2D_2Cl_4$, 333K): $\delta$ 8.60 (s, 8H); 8.41 (d, J = 8.2 Hz, 8H); 8.31(d, J = 8.2 Hz, 8H); 7.51 (d, J = 3.7 Hz, 8H); 7.33 (d, J = 3.7 Hz, 8H); 5.14-5.09 (m, 8H); 2.19-2.16 (m, 16H); 1.90-1.87 (m, 16H); 1.31-1.27 (m, 96H); 0.83-0.82(m, 48H). **$^{13}$C NMR** (100 MHz, $CDCl_3$): $\delta$ 164.31; 163.22; 144.16; 139.22; 135.91; 135.19; 134.04; 132.55; 132.21; 130.24; 129.69; 128.99; 128.41; 128.01; 125.69; 122.82; 122.24; 54.69; 32.22; 31.63; 26.50; 22.46; 13.93. **IR** (ATR-ZnSe) [cm $^{-1}$] 2923.3; 2855.9; 1696.9; 1655.9; 1584.6; 1454.6; 1401.4; 1320.7; 1241.7; 1179.6; 1118.9; 973.8; 926.6; 860.3; 832.7; 809.9; 755.6; 715.7. **HRMS** (MALDI, dithranol matrix) calculated m/z for $[C_{216}H_{224}N_8O_{16}S_8]^-$ 3441.4721; found 3441.4648.

**Pentamer**: **$^1$H NMR** (500 MHz, $CD_2Cl_2$): $\delta$ 8.59-8.57 (br, 10H); 8.35 (d, J = 8.2 Hz, 10H); 8.25 (br, 10H); 7.40 (d, J = 3.6 Hz, 10H); 7.35 (d, J = 3.6 Hz, 10H); 5.08 (br, 10H); 2.16-2.13 (m, 20H);

1.76-1.75 (m, 20H); 1.26-1.17 (m, 120H); 0.76-0.73 (m, 60H). **$^{13}$C NMR** (100 MHz, $CDCl_3$): $\delta$ 164.37; 163.28; 143.59; 139.25; 135.98; 135.26; 134.15; 132.69; 132.27; 130.33; 129.70; 129.09; 128.70; 128.10; 125.60; 123.02; 122.83; 122.30; 122.06; 54.63; 32.18; 31.63; 26.48; 22.45; 13.96. **IR** (ATR-ZnSe) [cm $^{-1}$] 2925.7; 2856.1; 1699.1; 1658.4; 1586.2; 1403.5; 1323.6; 1275.6; 1260.9; 750.7. **HRMS** (MALDI, dithranol matrix) calculated m/z for $[C_{270}H_{280}N_{10}O_{20}S_{10}]^-$ 4031.8402; found 4031.8465.

**Hexamer**: **$^1$H NMR** (500 MHz, $CDCl_3$): $\delta$ 8.62 (br, 12H); 8.36 (d, J = 8.2 Hz, 12H); 8.28 (br, 12H); 7.37-7.33 (m, 24H); 5.10 (br, 12H); 2.18-2.16 (m, 24H); 1.79-1.77 (m, 24H); 1.52-1.19 (m, 144H); 0.78-0.77 (m, 72H). ). **$^{13}$C NMR** (100 MHz, $CDCl_3$): $\delta$ 164.41; 163.28; 143.56; 139.26; 136.04; 135.35; 134.28; 132.89; 132.37; 131.20; 130.41; 129.74; 129.17; 128.74; 128.20; 127.95; 125.81; 122.93; 122.34; 54.72; 32.24; 31.67; 26.53; 22.48; 13.98. **IR** (ATR-ZnSe) [cm $^{-1}$] 2952.0; 2923.1; 2856.0; 1696.9; 1655.9; 1585.2; 1455.7; 1402.7; 1321.6; 1246.6; 1180.0; 1121.2; 862.4; 833.4; 757.8. **HRMS** (MALDI, dithranol matrix) calculated m/z for $[C_{324}H_{336}N_{12}O_{24}S_{12}]^-$ 5162.2084; found 5162.2105.

Interconversion study of both enantiomers of trimer **1**: The enantiomers of trimer **1** was separated on CHIRALPAK IA-3 column (21 mm ID x 250 mm, 5µm) on Waters Prep150 instrument eluting with 24% dichloromethane in hexane. Each enantiomer (1 mg each) labeled **trimer-entA** (shorter retention time, t = 14.7 min) and **trimer-entB** (longer retention time, t = 28.0 min) was dissolved in 1 mL of anhydrous 1,2-dichlorobenzene, sparged under $N_2$ for 30 minutes. It was placed in oil bath set at 160 $^oC$ for 19 hours. The solvent was distilled off under high vacuum reduced pressure. $^1$HNMR was identical to starting enantiomer and the chiral HPLC trace exhibited same retention time.

Synthesis of **1**-$Br_{12}$ (Supplementary Figure 23): A solution of trimer **1** (60 mg, 0.023 mmol) in $CH_2Cl_2$ (5 mL) was set stirring in a vial. Excess bromine (0.5 mL) was added, followed by a crystal of iodine. The reaction was capped and left to stir for 3 days. The solvent and bromine was purged under air. The resultant red solid was dissolved in chloroform and purified by small silica gel column chromatography, eluting with 100% chloroform to yield dark violet solids (65 mg, 0.018 mmol, 80% yield).

**$^1$H NMR** (400 MHz, $CDCl_3$): $\delta$ 8.77-8.74 (br, 6H); 8.36-8.33 (br, 6H); 8.07 (d, J = 8 Hz, 6H); 5.09-5.06 (m, 6H); 2.14-2.06 (m, 12H); 1.83 (br, 12H); 1.25 (br, 72H); 0.80-0.77 (m, 36H). **$^{13}$C NMR** (100 MHz, $CDCl_3$): $\delta$ 163.98; 163.61; 162.89; 162.48; 140.20; 135.36; 134.61; 133.31;

132.95; 130.86; 130.37; 129.52; 128.47; 128.07; 123.48; 122.78; 117.27; 116.26; 54.86; 32.07; 31.56; 26.51; 22.45; 13.94. **IR** (ATR-ZnSe) [cm $^{-1}$] 3005.9; 2986.5; 2923.4; 2851.9; 1696.9; 1656.4; 1584.9; 1394.5; 1320.0; 1260.8; 862.9; 811.9; 750.5. **HRMS** (MALDI, dithranol matrix) calculated m/z for $[C_{162}H_{156}Br_{12}N_6O_{12}S_6]^-$ 3516.0306; found 3516.0356

## Supplementary References


1. Rajasingh, P., Cohen, R., Shirman, E., Shimon, L.J. & Rybtchinski, B. Selective bromination of perylene diimides under mild conditions. *J. Org. Chem.* **72**, 5973-5979 (2007).
2. CrysAlisPRO, O.D. Agilent Technologies UK Ltd. *Yarnton, England Search PubMed* (2011).
3. Sheldrick, G. A short history of SHELX. *Acta Crystallogr., Sect. A* **64**, 112-122 (2008).
4. Sheldrick, G. Crystal structure refinement with SHELXL. *Acta Crystallogr., Sect. C* **71**, 3-8 (2015).
5. Palatinus, L. & Chapuis, G. SUPERFLIP - a computer program for the solution of crystal structures by charge flipping in arbitrary dimensions. *J. Appl. Crystallogr.* **40**, 786-790 (2007).
6. Dolomanov, O.V., Bourhis, L.J., Gildea, R.J., Howard, J.A.K. & Puschmann, H. OLEX2: a complete structure solution, refinement and analysis program. *J. Appl. Crystallogr.* **42**, 339-341 (2009).
7. Spek, A. Structure validation in chemical crystallography. *Acta Crystallographica Section D* **65**, 148-155 (2009).
8. Sheldrick, G. Phase annealing in SHELX-90: direct methods for larger structures. *Acta Crystallogr., Sect. A* **46**, 467-473 (1990).
9. Le, Y. MISSYM1.1 - a flexible new release. *J. Appl. Crystallogr.* **21**, 983-984 (1988).
10. Betteridge, P.W., Carruthers, J.R., Cooper, R.I., Prout, K. & Watkin, D.J. CRYSTALS version 12: software for guided crystal structure analysis. *J. Appl. Crystallogr.* **36**, 1487 (2003).
11. Hammersley, A.P., Svensson, S.O., Hanfland, M., Fitch, A.N. & Hausermann, D. Two-dimensional detector software: From real detector to idealised image or two-theta scan. *High Pressure Res.* **14**, 235-248 (1996).
12. Yang, X., Juhás, P., Farrowa, C.L. & Billinge, S.J.L. PDFgetXgui: a graphical user interface for PDFgetX3 for high throughput pair distribution function transformation and visualization. *ArXiv* **V2** (2014).
13. Juhás, P., Davis, T., Farrow, C.L. & Billinge, S.J. PDFgetX3: a rapid and highly automatable program for processing powder diffraction data into total scattering pair distribution functions. *J. Appl. Crystallogr.* **46**, 560-566 (2013).
14. Toby, B. & Egami, T. Accuracy of pair distribution function analysis applied to crystalline and non-crystalline materials. *Acta Crystallogr., Sect. A: Found. Crystallogr.* **48**, 336-346 (1992).
15. Qiu, X., Božin, E.S., Juhas, P., Proffen, T. & Billinge, S.J. Reciprocal-space instrumental effects on the real-space neutron atomic pair distribution function. *J. Appl. Crystallogr.* **37**, 110-116 (2004).
16. Farrow, C. *et al.* PDFfit2 and PDFgui: computer programs for studying nanostructure in crystals. *J. Phys.: Condens. Matter* **19**, 335219 (2007).
